# Identification of highly potent **α**-glucosidase inhibitory and antioxidant constituents from *Zizyphus rugosa* bark: enzyme kinetic and molecular docking studies with active metabolites

**DOI:** 10.1080/13880209.2017.1304426

**Published:** 2017-03-21

**Authors:** Jirapast Sichaem, Thammarat Aree, Kiattisak Lugsanangarm, Santi Tip-pyang

**Affiliations:** a Natural Products Research Unit, Department of Chemistry, Faculty of Science, Chulalongkorn University, Bangkok, Thailand;; b Program of Chemistry, Faculty of Science and Technology, Bansomdej Chaopraya Rajabhat University, Bangkok, Thailand

**Keywords:** Lupane-type triterpenes, lignan glycosides, flavonoid glycosides

## Abstract

**Context:** Previous studies have shown that extracts of *Zizyphus rugosa* Lam. (Rhamnaceae) bark contained phytoconstituents with antidiabetic potential to lower blood glucose levels in diabetic rats. However, there has been no report on the active compounds in this plant as potential antidiabetic inhibitors.

**Objective:** We evaluated the *α*-glucosidase inhibitory and antioxidant activities of *Z. rugosa* extract. Moreover, the active phytochemical constituents were isolated and characterized.

**Materials and methods:** The *α*-glucosidase inhibition of crude ethanol extract obtained from the bark of *Z. rugosa* was assayed as well as the antioxidant activity. Active compounds (**1–6**) were isolated, the structures were determined, and derivatives (**2a–2 l**) were prepared. All compounds were tested for their α-glucosidase inhibitory (yeast and rat intestine) and antioxidant (DPPH) activities.

**Results:** The active α-glucosidase inhibitors (**1–6**) were isolated from *Z. rugosa* bark and 12 derivatives (**2a–2 l**) were prepared. Compound **2** showed the most powerful yeast α-glucosidase inhibitory activity (IC_50_ 16.3 μM), while compounds **3** and **4** display only weak inhibition toward rat intestinal *α*-glucosidase. Moreover, compound **6** showed the most potent antioxidant activity (IC_50_ 42.8 μM). The molecular docking results highlighted the role of the carboxyl moiety of **2** for yeast α-glucosidase inhibition through H-bonding.

**Discussion and conclusions**: These results suggest the potential of *Z. rugosa* bark for future application in the treatment of diabetes and active compounds **1** and **2** have emerged as promising molecules for therapy.

## Introduction

Diabetes mellitus (DM) is a group of metabolic disorders in which there are high blood glucose levels over a prolonged period (Palanisamy et al. 2011). Type 2 diabetes mellitus (T2DM) is a typical chronic metabolic disorder characterized by hyperglycaemia in the context of insulin resistance (Kitabchi et al. 2009). Among several drug types available, α-glucosidase inhibitors have been marketed for treatment of T2DM. Most can control diabetes and its complications by suppressing blood glucose levels. So far, several α-glucosidase inhibiting drugs have been developed from natural prototypes (Moorthy et al. 2012).

*Zizyphus rugosa* Lam. (Rhamnaceae), locally known as ‘Ma Khwat’, is a large straggling shrub with recurved prickles mainly distributed in Laos, China (Hainan, Yunnan), India, Burma, Sri Lanka, Vietnam and Thailand. Previous phytochemical studies on this plant revealed various types of compounds, including flavonoids, alkaloids and triterpenoids (Kaennakam et al. 2013). Traditionally, its root and stem bark, leaves and flowers are used in the preparation of herbal formulations (Krishnamurthy & Sarala 2012). Several studies reported that this plant shows analgesic, anti-inflammatory, antibacterial, antifungal, anxiolytic, CNS depressant, cytotoxic, antimicrobial and antioxidant properties (Prashith Kekuda et al. 2011; Rambabu et al. 2011; Mohamad et al. 2013). Interestingly, it has been previously reported that the *Z. rugosa* bark extracts contain phytoconstituents with antidiabetic potential to lower blood glucose levels in diabetic rats (Marles & Farnsworth 1995; Mohamad et al. 2013). However, there has been no report on the identification of active compounds in this plant as potential antidiabetic inhibitors. This study was therefore carried out to search for active α-glucosidse inhibitory and antioxidant compounds from the bark of this plant. In addition, the enzyme kinetic activity, including the binding conformations and important interactions between potent inhibitors and *α*-glucosidse was investigated.

## Materials and methods

### General experimental procedures

1 D and 2 D NMR spectra were recorded on a Bruker 400 AVANCE spectrometer, and the chemical shifts were reported in parts per million (ppm) using tetramethylsilane (TMS) as the internal standard. Melting points were determined on a Fisher-Johns Melting Point apparatus. Adsorbents such as silica gel 60 (Merck) were used for column and radial (chromatotron model 7924 T, Harrison Research) chromatographies. Merck silica gel 60F_254_ plates were used for TLC. HRESIMS spectra were obtained using a Bruker MICROTOF model mass spectrometer. UV-visible absorption spectra were recorded using a UV-2550 (SHIMADZU) UV-Vis spectrometer (Shimadzu, Kyoto, Japan). α-Glucosidase (EC 3.2.1.20) from *Saccharomyces cerevisiae* and 4-nitrophenyl-α-D-glucopyranoside (*p-*NPG) were obtained from Sigma-Aldrich (St. Louis, MO). Acarbose was obtained from Bayer Vitol Leverkusen, Germany. Rat intestinal acetone powder was supplied by Sigma Aldrich.

### Plant material

The bark of *Z. rugosa* was collected from Mahasarakham province of Thailand in November 2014. The plant material was identified by Dr. Suttitra Khumkratok, a botanist at Walai Rukhavej Botanical Research Institute, Mahasarakham University, where a voucher specimen (khumkratok no. 1-13) is deposited.

### Extraction and isolation

The air-dried bark of *Z. rugosa* (1.7 kg) was macerated with 4 L of EtOH at room temperature for five days. The solvent was evaporated to afford crude EtOH extract (178.0 g). This crude extract was subjected to vacuum liquid chromatography (VLC) over silica gel (Merck Art 7730), using successive eluents of hexane, CH_2_Cl_2_, EtOAc and MeOH, with increasing polarity to afford seven major fractions (A-G) which were then evaluated for their α-glucosidase inhibitory and antioxidant activities (Table 1). Based on the primary screening results, the active fraction D was further purified by column chromatography over silica gel using hexane-EtOAc (100:0-0:100) and MeOH as an eluent to afford three subfractions (D1-D3). Subfractions D1 and D3 were further purified using a combination of silica gel column and radial (chromatotron) chromatographies, affording two major triterpenoids (**1**, 0.51 g and **2**, 1.94 g). Finally, active fraction G was isolated by silica gel column chromatography and eluting with CH_2_Cl_2_-MeOH (100:0-0:100) to yield three subfractions (G1-G3). Subfraction G2 was selected for purification by Sephadex LH-20 (2:8 MeOH–CH_2_Cl_2_) followed by RP-HPLC (ODS, 50:50 MeOH–H_2_O, UV 254 nm) to furnish **3** (2.5 mg, t_R_ 32.4 min) and **4** (4.4 mg, t_R_ 30.5 min). Subfraction G3 was subsequently purified by silica gel column chromatography (CH_2_Cl_2_-MeOH (90:10-0:100)) followed by Sephadex LH-20 (3:7 MeOH–CH_2_Cl_2_) to give **5** (4.3 mg) and **6** (2.1 mg). Their structures were identified by interpretation of their spectroscopic data (1 D and 2 D NMR and MS) as well as comparison with those reported in the literature.

**Table 1.** *α-*Glucosidase inhibitory and antioxidant (DPPH) activities of fractions A-G.

	IC_50_ (mg/mL)			
Fraction	Baker’s yeast	Maltase	Sucrase	DPPH
A	0.27 ± 1.41	>1.0	>1.0	>10.0
B	0.19 ± 0.89	>1.0	>1.0	>10.0
C	0.081 ± 0.67	>1.0	>1.0	8.76 ± 1.49
D	0.052 ± 1.23	>1.0	>1.0	2.44 ± 2.16
E	0.094 ± 0.59	>1.0	>1.0	1.47 ± 0.37
F	0.43 ± 1.75	>1.0	>1.0	0.13 ± 0.11
G	0.14 ± 1.22	0.12 ± 0.57	>1.0	0.025 ± 1.85
Acarbose	0.34 ± 1.59	0.0051 ± 0.94	0.0071 ± 2.36	–
Vitamin C	–	–	–	0.028 ± 1.56
BHT	–	–	–	0.072 ± 0.35

### General procedure for modification of derivatives (2a-2f)

Briefly, a compound **2** in CH_2_Cl_2_ was added triethylamine (TEA), 4-(dimethylamino) pyridine (DMAP) and various anhydrides. The solution was stirred at room temperature for 1 h. The reaction was diluted by CH_2_Cl_2_, washed with brine and dried over anhydrous Na_2_SO_4_. After removal of solvent, the residue was purified on a chromatotron to give their derivatives. For more details were described in Supporting Information.

### α*-Glucosidase inhibition assay*

The evaluation of the inhibitory activity of all compounds against yeast and rat intestinal (maltase and sucrase) α-glucosidases was applied from a previous procedure (Damsud et al. 2013). The inhibitory effect against yeast α-glucosidase was determined using the protocol previously described. Briefly, 10 μL of sample was mixed with α-glucosidase (0.1 U/mL) in 1 mM phosphate buffer (pH 6.9) and incubated at 37 °C for 10 min. The reaction mixture was added with 1 mM *p*-nitrophenyl-α-D-glucopyranoside (PNPG, 50 μL) followed by additional 20 min incubation. *p*-Nitrophenoxide liberated from the reaction was quantified by a microplate reader (UV 405 nm) after the addition of 1 M Na_2_CO_3_ (100 μL). The percent inhibition was deduced using the following equation.

$$\begin{array}{cc} \frac{\left({\text{A}}_{\text{0}}-{\text{A}}_{\text{1}}\right)}{{\text{A}}_{\text{0}}}\times 100 & \end{array}$$

where A_1_ and A_0_ are absorbances with and without the sample, respectively.

Moreover, previous procedure (Damsud et al. 2013) was also applied to determine inhibitory activity of isolated compounds against intestinal α-glucosidases (maltase and sucrase). Briefly, 10 μL of the test sample and substrate solution (maltose: 10 mM, 20 μL; sucrose: 100 mM, 20 μL, respectively) in 0.1 M phosphate buffer (pH 6.9) were incubated at 37 °C (20 min for maltase and 60 min for sucrase). The mixture was discontinued in boiling water for 10 min, and glucose released from the reaction was converted to quinoneimine using a commercial Glu Kit (Human, Germany). The absorbance of final product was determined at 503 nm, and percent inhibition was calculated using the aforementioned equation.

### DPPH radical scavenging activity

The antioxidant activity of the extract was evaluated through the free radical scavenging effect on 2,2*′*-diphenyl-1-picrylhydrazyl (DPPH) (Sigma) radical as previously reported (Yen & Hsieh 1997) with slight modifications. 100 μL of 0.10 mM DPPH ethanolic solution was added to 20 μL of the sample with various concentrations. The mixture was thoroughly mixed and kept in the dark for 30 min. The absorbance was measured at 517 nm using a Sunrise microplate reader spectrophotometer.

### Methods of computational analysis

#### Protein homology modelling and inhibitor preparation

The amino acid sequence of the yeast α-glucosidase was obtained from the Gen Bank database (GenBank CAA00532.1) (Lee et al. 2014). The BLAST search module of Discovery Studio version 2.0 (Accelrys) was used to search for the most similar three-dimensional crystal structure with yeast α-glucosidase.

It was found that the crystal structure with the pdb code of 3A4A, which has the X-ray crystallographic resolution of 1.60 Å, showed the highest identity and similarity percentage of 71.3 and 86.7, respectively (Yamamoto et al. 2010). Therefore, the pdb code: 3A4A was further used as the template for yeast α-glucosidase structure.

The Protein Modelling module of Discovery Studio version 2.0 was used to determine the three-dimensional structure of yeast α-glucosidase (Lugsanangarm et al. 2011). Ten satisfactory protein models were constructed and the justification of quality was based on the density optimization potential energy (DOPE) score (Lugsanangarm et al. 2011). The structure which showed a lowest DOPE score was selected for further study. Subsequently, the homology modelled structure was subjected for energetic relaxation using the Amber14 software package with the amber14sb force field (Case et al. 2008). The protein was energetically minimized with 1000 steps using steepest descent minimization, followed by another 2000 steps of conjugate gradient minimization. Subsequently, the protein was then solvated by TIP3P water molecules extending to 12 Å in each of the six directions: (±*x*, ±*y*, and ±*z*) around the protein. The periodic boundary condition was employed. Appropriate sodium counter ions were then added to neutralize the systems. Subsequently, 2000 steps of steepest descent minimization were performed on the whole system followed by 3000 steps of conjugate gradient minimization. This minimized system was used as the starting structure for the subsequent molecular docking calculations.

Compounds **1** and **2** were constructed and were energetically optimized using the MOPAC2009 program (James 2008). These optimized structures were further used in molecular docking calculation.

#### Molecular docking calculation

Molecular docking calculations were performed for compounds **1** and **2** using the AutoDock 4.2.6 program (Morris et al. 2009). The grid box was defined as 100 × 100 × 100 points as reported (Peng et al. 2016). It covered the amino acid residue of Asp69, Phe178, Thr215, Glu277, His351, Asp352, Arg315, Phe314 and Val410. These amino acids are believed to play critical roles in the catalytic mechanism of α-glucosidase. The 500 running numbers and Lamagian GA algorithm were employed while other parameters were set as default.

#### Molecular interaction analysis

The docking poses which displayed the lowest binding free energies were selected for molecular interaction analyses. The molecular conformation of compounds **1** and **2** were analyzed and depicted using Chimera software (Pettersen et al. 2004). Moreover, the hydrophobic interactions have been analyzed by using the LigPlot^+^ software (Cambridge, United Kingdom) (Laskowski & Swindells 2011). 

## Results

### Bio-assay guided extraction and isolation

**Figure 1.**
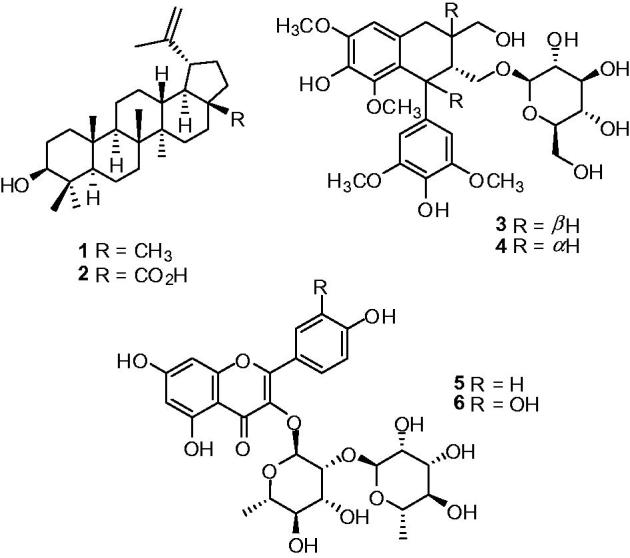
Structures of isolated compounds (**1-6**) from active fractions D and G of *Z. rugosa*.

As described previously, the crude EtOH extract (178.0 g) obtained from the dried bark of *Z. rugosa* (1.7 kg) was fractionated to yield seven main fractions (A-G). Then, their inhibitory effects against α-glucosidases from Baker's yeast and rat intestine together with antioxidant activity were examined. Based on the bio-assay guide (Table 1), fraction D revealed the most potent activity toward baker's yeast α-glucosidase (IC_50_ 0.052 mg/mL), while fraction G showed significant but weaker activities toward α-glucosidase from yeast (IC_50_ 0.14 mg/mL) and intestine (maltase) (IC_50_ 0.12 mg/mL), respectively. In addition, fraction G also exhibited potent antioxidant activity with the IC_50_ value of 0.025 mg/mL. Therefore, fractions D and G were selected for further isolation and afforded two triterpenoids, lupeol (**1**) and betunilic acid (**2**) (Kaennakam et al. 2013) from fraction D, two lignan glycosides, (6 *S*,7 *R*,8 *R*)-7*α*-[(*β-*glucopyranosyl)oxy] lyoniresinol (**3**) and (+)-lyoniresinol-*3α-O-β*-d-glucopyranoside (**4**) (Ohashi et al. 1994) as well as two flavonoid glycosides, kaempferol-3-*O*-α-l-rhamnopyranosyl-(1→2)-α-l-rhamnopyrano side (**5**) and horridin (**6**) (Olennikov & Kashchenko 2013) from fraction G (Figure 1).

### α-Glucosidase activity and kinetic study

All isolated compounds (**1–6**) were evaluated for their baker's yeast and rat intestinal α-glucosidase activities (Table 2). Among these compounds, compounds **1** and **2** exhibited potential yeast α-glucosidase activity with the IC_50_ values of 37.2 and 16.3 μM, respectively, which were superior to the positive drug (acarbose) (IC_50_ 526 μM). On the other hand, compounds **3** and **4** (from fraction G) display only weak inhibitions toward rat intestinal *α*-glucosidase (maltase and sucrase) with IC_50_ values of 1,480 and 2,341 μM, and 373 and 1,214 μM, respectively.

**Table 2.** *α-*Glucosidase inhibitory and antioxidant (DPPH) activities of **1–6** and **2a–2l**.

	IC_50_ (μM)			
Compound	Baker’s yeast	Maltase	Sucrase	DPPH
**1**	37.2 ± 0.22	>3000	>3000	>10,000
**2**	16.3 ± 2.03	>3000	>3000	>10,000
**3**	>600	1480 ± 2.13	2341 ± 1.43	4321 ± 1.13
**4**	>600	373 ± 1.05	1214 ± 2.01	3795 ± 0.42
**5**	>600	>3000	>3000	6019 ± 1.36
**6**	>600	>3000	>3000	42.8 ± 0.83
**2a**	28.1 ± 0.76	>3000	>3000	>10,000
**2b**	43.3 ± 0.19	>3000	>3000	>10,000
**2c**	158 ± 1.22	>3000	>3000	>10,000
**2d**	269 ± 3.22	>3000	>3000	>10,000
**2e**	72.1 ± 1.54	>3000	>3000	>10,000
**2f**	73.1 ± 0.96	>3000	>3000	>10,000
**2g**	230 ± 0.11	>3000	>3000	>10,000
**2h**	281 ± 0.89	>3000	>3000	>10,000
**2i**	368 ± 1.64	>3000	>3000	>10,000
**2j**	>600	>3000	>3000	>10,000
**2k**	>600	>3000	>3000	>10,000
**2l**	>600	>3000	>3000	>10,000
Acarbose	526 ± 1.52	7.9 ± 0.94	10.9 ± 2.36	–
Vitamin C	–	–	–	158 ± 1.90
BHT	–	–	–	328 ± 0.56

**Scheme 1.**
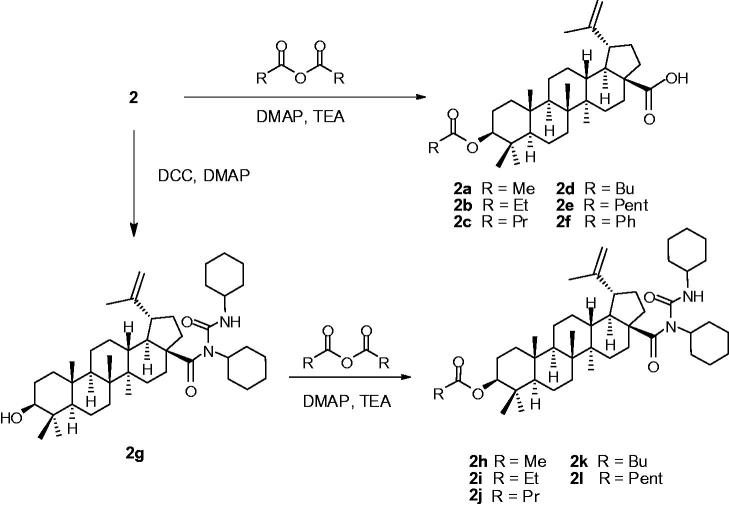
Modified reactions of betunilic acid (**2**).

In this study, compound **2** exhibited the most powerful inhibitory activity against yeast α-glucosidase. This result drew our interest to prepare the derivatives (**2a–2 l**) in the course of chemical modifications (Scheme 1). All derivatives (**2a–2 l**) were also evaluated for their α-glucosidase activity (Table 2). Compounds **2a**, **2 b**, **2e** and **2f** exhibited potential yeast α-glucosidase activity with the IC_50_ values in the range of 28.1 to 73.1 μM. In addition, compounds **2c**, **2d**, and **2g–2i** display good inhibitions toward yeast *α*-glucosidase with the IC_50_ values in the range of 158 to 368 μM, which were also superior to the positive drug (acarbose) (IC_50_ 526 μM).

**Figure 2.**
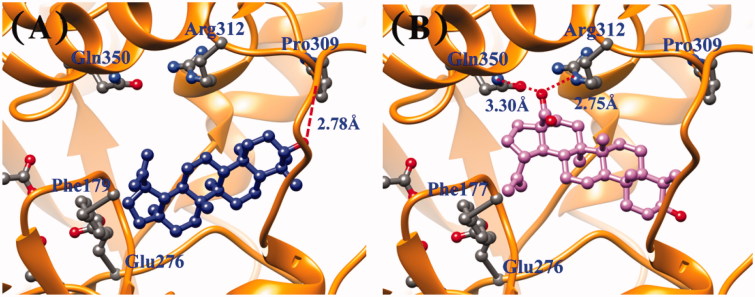
Comparison of molecular binding pose of compounds **1** (A) and **2** (B) in yeast α-glucosidase.

The Lineweaver–Burk plots of a kinetic study of **2** showed linearity at each concentration examined (0.063 and 1.3 mM); all of which intersected at the second quadrant. The kinetic analysis revealed that *V*_max_ decreased with increasing concentrations of **2** while *K*_m_ increased (Figure 2S). This behaviour indicated that **2** inhibited as a mixed mode *inhibitor* of α-glucosidase enzyme in which noncompetitive mode (*K_i_* 11.16 mM, Figure 3S) was preferred over competitive manner (*K'_i_* 0.019 mM, Figure 4S).

### Antioxidant activity

All isolated compounds (**1–6**) and derivatives (**2a–2l**) were also evaluated for their DPPH free radical scavenging activity (Table 2). Compound **6** showed the most potent antioxidant activity with the IC_50_ value of 42.8 μM and indeed, this was stronger than the reference compounds (vitamin C and BHT). Compounds **3–5** likewise were weakly active (IC_50_ 4,321, 3,795 and 6,019 μM). By contrast, other compounds (**1** and **2**) showed no activity.

### Molecular docking studies

To evaluate the activity of lupane-type triterpenoids (**1** and **2**) against yeast α-glucosidase, molecular docking calculations were employed. Figure 2 shows the molecular binding of **1** and **2**. Compound **1** revealed the binding free energy of −5.83 kcal/mol while compound **2** exhibited −6.11 kcal/mol. These energies were in accordance with the experimental IC_50_ values [compounds **1** (0.037 mM) and **2** (0.016 mM)]. It was also found that compound **1** showed hydrogen bonding (H-bond) between the hydroxyl part and O (backbone) of Pro309 with the distance of 2.78 Å. This H-bond disappeared in **2**. The two H-bonds, however, were formed between the oxygen atom of the carboxyl group of **2** and N (side-chain) of Arg312 and O (side-chain) of Gln350, with the distance of 2.75 and 3.30 Å, respectively.

The hydrophobic interaction analyses revealed that compound **1** had hydrophobic interaction with Phe157, Phe178, Thr215, Leu218, Asn241, His245, Glu276, Ala278, His279 and Glu304, while compound **2** showed hydrophobic interaction with Asn241 and His245, Glu276, His279, Phe300 and Glu304 as depicted in Figure 5S.

## Discussion

The selected active fractions D and G were isolated and obtained two triterpenoids (**1** and **2**) from fraction D, two lignan glycosides (**3** and **4**) and two flavonoid glycosides (**5** and **6**) from fraction G. All isolated compounds (**1–6**) were evaluated for their α-glucosidase activity, compound **2** showed most powerful inhibitory activity against yeast α-glucosidase. The above result motivated our group to investigate the structural modification and α-glucosidase inhibitory activity of their derivatives (**2a–2l**) from that natural lupine-type (**2**).

In the course of chemical modifications, the series of analogues of **2** containing the modifications of C-3 and C-28 positions were prepared to yield seven new (**2b** and **2g–2l**) and five known compounds (**2a** and **2c–2f**). In the derivatization of **2**, the amidation of **2** with DCC obtained a key intermediate (**2g**) and proved its structure by using single crystal X-ray crystallography (Figure 1S), followed by nucleophilic acylation of the hydroxyl group of **2g** with appropriate anhydrides to afford a new series of C-3 amide ester derivatives (**2h–2l**). All derivatives (**2a–2l**) were also evaluated for their α-glucosidase activity (Table 2). Compounds **2a–2f**, which lacked the hydroxyl group at C-3, showed lower activities than their originals (**2**) toward α-glucosidase. It is noteworthy that the hydroxyl group appeared to be involved in the yeast α-glucosidase inhibitory activity of **2**. In addition, the enhancement of the side chain hydrophobicity at C-3 led to a decrease in antidiabetic activity. On the other hand, compound **2g**, which lacked the carboxyl group at C-28, showed lower IC_50_ values against α-glucosidase than its original (**2**). Therefore, our finding enlightened us that the carboxyl group might be the pivotal functional group in yeast α-glucosidase inhibitory activity of **2**. To envision the mechanism underlying this inhibition, a kinetic study of this potent metabolite (**2**) was performed. The Lineweaver-Burk plots indicated that **2** inhibited as a mixed mode inhibitor of α-glucosidase enzyme in which noncompetitive mode was preferred over competitive manner. The results of this study suggested the potential of natural compounds **1** and **2** as lead compounds for the developing antidiabetic agents; their antidiabetic properties may be related to their selectively inhibitory on Type I α-glucosidase (Baker’s yeast).

All isolated compounds (**1–6**) and derivatives (**2a–2l**) were also evaluated for their antioxidant activity. Compound **6** showed the most potent antioxidant activity and stronger than the reference agents while compounds **3–5** were weakly active. These results suggested that the greater effectiveness of **6** with *o-*dihydroxy group at C-3′ and C-4′ was possibly due to the presence of the catechol group which, upon donating hydrogen radicals, gives a higher stability to their radical forms (Udomchotphruet et al. 2012).

In the molecular docking calculations, compound **1** showed hydrogen bonding (H-bond) between the hydroxyl part and O (backbone) of Pro309. This H-bond disappeared in **2**. The two H-bonds, however, were formed between the oxygen atom of the carboxyl group of **2** and N (side-chain) of Arg312 and O (side-chain) of Gln350. The H-bond with Arg312 energetic preferably interacted due to the negative oxygen and positively charged Arg312. This polar interaction is also reported (Ma et al. 2015; Jabeen et al. 2016) and suggesting that Arg312 may has catalytic role in the inhibition of enzyme function. (Ma et al. 2015; Jabeen et al. 2016). The –C(CH_3_)=CH_2_ group of **1** was placed far from Gln350 and Arg312 approximately of 7.5 Å. Due to a large pocket, compound **2** could flip, and this moiety pointed toward Thr215. This pose allowed H-bond forming between the carboxyl group and Arg312 and Gln350, as mentioned above. These results highlighted the role of carboxyl moiety at the C-28 position for yeast α-glucosidase inhibition through the H-bond.

## Conclusions

This is the first report on mainly active α-glucosidase inhibitors and antioxidant metabolites present in the bark of *Z. rugosa*. Six compounds (**1–6**) were isolated based on bio-assay guided isolation and twelve derivatives (**2a–2l**) were prepared. Compound **2** showed the most powerful yeast α-glucosidase inhibitory activity, which was superior to a positive agent. On the other hand, compounds **3** and **4** exhibited weak inhibitory activity against rat intestinal α-glucosidase. In addition, compound **6** exhibited the most potent antioxidant (DPPH) activity. A subsequent investigation on the mechanism underlying the inhibitory effect of compound **2** suggested that it blocked yeast α-glucosidase by mixed inhibition *via* competitive and noncompetitive manners. Moreover, the molecular docking studies of **1** and **2** on the binding sites of yeast α-glucosidase were performed in order to afford a molecular insight into the mode of action of these active compounds. Therefore, this study can help to support the traditional use of *Z. rugosa* as an antidiabetic remedy.

## Supplementary Material

Santi_Tip-Pyang_et_al_supplemental_content.zip
