# Supplementary material for: Identification of highly potent α-glucosidase inhibitory and antioxidant constituents from Zizyphus rugosa bark: enzyme kinetic and molecular docking studies with active metabolites
Source: Pharm Biol. 2017 Mar 21;55(1):1436–41. doi: 10.1080/13880209.2017.1304426 (PMC6130710; doi:10.1080/13880209.2017.1304426)
Supplement: Santi_Tip-Pyang_et_al_supplemental_content.zip [file IPHB_A_1304426_SM2415.zip › Santi Tip-Pyang et al supplemental content.pdf]

SUPPORTING INFORMATION

Identification of highly potent *α*-glucosidase inhibitory and antioxidant constituents from *Zizyphus rugosa* bark: enzyme kinetic and molecular docking studies on active metabolites

Jirapast Sichaem[a], Kiattisak Lugsanangarm[b] and Santi Tip-pyang[a,*]

[a]*Natural Products Research Unit, Department of Chemistry, Faculty of Science, Chulalongkorn University, Bangkok, 10330, Thailand*

[b]*Program of Chemistry, Faculty of Science and Technology, Bansomdej Chaopraya Rajabhat University, Bangkok, 10600, Thailand*

* Corresponding author. Tel.: + 66 2 218 7625. E-mail address: Santi.ti@chula.ac.th

Keywords: lupane-type triterpenoids, lignan glycosides, flavonoid glycosides

General procedure for modification of derivatives (2a-2f)

Briefly, a compound **2** in CH_2Cl_2 was added triethylamine (TEA), 4-(dimethylamino) pyridine (DMAP) and various anhydrides. The solution was stirred at room temperature for 1 h. The reaction was diluted by CH_2Cl_2, washed with brine and dried over anhydrous Na_2SO_4. After removal of solvent, the residue was purified on a chromatotron to give their derivatives. For more details were described following;

3-O-Acetyl betulinic acid (2a): Following the general procedure, reaction of **2** (50.0 mg, 0.11 mmol), acetic anhydride (31 µL, 0.35 mmol), TEA (46 µL, 0.33 mmol), DMAP (trace amount) in CH_2Cl_2 (1.09 mL) after 1 h yielded **2a** (24.1 mg, 44.1%) (Ahmad et al., 2010).

3-O-Propanoybetulinic acid (2b): Following the general procedure, reaction of **2** (50.0 mg, 0.11 mmol), propionic anhydride (42 µL, 0.35 mmol), TEA (46 µL, 0.33 mmol), DMAP (trace amount) in CH_2Cl_2 (1.09 mL) after 1 h yielded **2b** (29.7 mg, 52.9%). Compound **2b**: White Gum; ^1H NMR ($CDCl_3$, 400 MHz) δ_H 5.30 (1H, s), 4.74 (1H, s), 4.11 (1H, t, *J* = 8.0 Hz), 3.02 (1H, m), 2.28 (1H, m), 2.28 (2H, m), 2.21 (1H, m), 1.75 (1H, m), 1.74 (1H, m), 1.74 (1H, m), 1.69 (3H, s), 1.64 (1H, m), 1.62 (1H, m), 1.56 (1H, m), 1.56 (1H, m), 1.48 (1H, m), 1.46 (1H, m), 1.43 (1H, m), 1.40 (1H, m), 1.39 (1H, m), 1.38 (1H, m), 1.32 (1H, m), 1.25 (3H, m), 1.09 (1H, m), 1.08 (3H, m), 1.07 (1H, m), 1.07 (1H, m), 1.01 (1H, m), 1.00 (6H, s), 0.97 (3H, s), 0.97 (1H, m), 0.93 (3H, s), 0.90 (3H, s). ^{13}C NMR ($CDCl_3$, 100 MHz) δ_C 181.5, 174.5, 150.5, 109.9, 80.8, 56.5, 55.6, 50.6, 49.5, 47.1, 42.6, 40.9, 38.6, 38.6, 38.1, 37.3, 37.2, 34.4, 32.3, 29.9, 29.8, 28.2, 28.1, 25.6, 23.9, 21.0, 19.5, 18.3, 16.7, 16.3, 16.2, 14.8, 9.5. ESI *m/z* [M+Na$^+$] calcd for $C_{34}H_{54}O_4Na$: 549.4; found 549.4.

3-O-Butanoyl betulinic (2c): Following the general procedure, reaction of **2** (50.0 mg, 0.11 mmol), butanoic anhydride (54 µL, 0.35 mmol), TEA (46 µL, 0.33 mmol), DMAP (trace amount) in CH_2Cl_2 (1.09 mL) after 1 h yielded **2c** (24.8 mg, 43.0%) (Ahmad et al., 2010).

3-O-Pentanoyl betulinic acid (2d): Following the general procedure, reaction of **2** (50.0 mg, 0.11 mmol), valeric anhydride (66 µL, 0.35 mmol), TEA (46 µL, 0.33 mmol), DMAP (trace amount) in CH_2Cl_2 (1.09 mL) after 1 h yielded **2d** (30.1 mg, 50.8%) (De et al., 2012).

3-O-Hexanoyl betulinic acid (2e): Following the general procedure, reaction of **2** (50.0 mg, 0.11 mmol), hexanoic anhydride (76 µL, 0.35 mmol), TEA (46 µL, 0.33 mmol), DMAP

(trace amount) in CH_2Cl_2 (1.09 mL) after 1 h yielded **2e** (27.3 mg, 44.9%) (Zhang et al., 2010).

3-O-Benzoyl betulinic acid (2f): Following the general procedure, reaction of **2** (50.0 mg, 0.11 mmol), benzoic anhydride (66 mg, 0.35 mmol), (TEA (46 µL, 0.33 mmol), DMAP (trace amount) in CH_2Cl_2 (1.09 mL) after 1 h yielded **2f** (26.4 mg, 43.0%) (De et al., 2012).

General procedure for modification of derivatives (2g-2l)

Briefly, a compound **2** in CH_2Cl_2 was added *N,N'*-dicyclohexylcarbodiimide (DCC) and DMAP. The solution was stirred at room temperature for 1 h. After removal of solvent, the residue was purified on a chromatotron to give an intermediate (**2g**). This intermediate in CH_2Cl_2 was added TEA, DMAP and various anhydrides. The solution was stirred at room temperature for 1 h. The reaction was diluted by CH_2Cl_2, washed with brine and dried over anhydrous Na_2SO_4. After removal of solvent, the residue was purified on a chromatotron to give their derivatives. For more details were described following;

28-N-Cyclohexyl-N-[(cyclohexylamino)carbonyl]betulinic acid (2g): Following the general procedure, reaction of **2** (150.0 mg, 0.02 mmol), DCC (90 µL, 0.05 mmol), DMAP (trace amount) in CH_2Cl_2 (1.5 mL) after 1 h yielded **2g** (63.5 mg, 42.4%). Compound **2g**: White Solid; mp. 193-194 °C; ^{1}H NMR ($CDCl_3$, 400 MHz) δ_H 6.34 (1H, br, NH), 4.71 (1H, s), 4.57 (1H, s), 4.06 (1H, m), 4.04 (1H, br), 3.66 (1H, br), 3.11 (1H, m), 2.41 (1H, m), 2.20 (1H, m), 2.01 (1H, m), 1.90 (1H, m), 1.82 (1H, m), 1.77 (2H, m), 1.69 (1H, m), 1.68 (3H, s), 1.67 (2H, m), 1.65 (2H, m), 1.65 (2H, m), 1.64 (2H, m), 1.63 (1H, m), 1.61 (2H, m), 1.60 (3H, m), 1.59 (2H, m), 1.56 (1H, m), 1.55 (2H, m), 1.50 (1H, m), 1.48 (1H, m), 1.45 (1H, m), 1.44 (1H, m), 1.40 (1H, m), 1.38 (2H, m), 1.37 (1H, m), 1.36 (2H, m), 1.34 (1H, m), 1.27 (1H, m), 1.26 (1H, m), 1.25 (1H, m), 1.20 (1H, m), 1.19 (2H, m), 1.18 (2H, m), 1.17 (2H, m), 1.16 (2H, m), 1.15 (1H, m), 1.14 (1H, m), 1.09 (2H, m), 1.08 (1H, m), 1.02 (2H, m), 1.01 (1H, m), 0.97 (3H, s), 0.95 (3H, s), 0.91 (3H, s), 0.82 (3H, s), 0.81 (2H, m), 0.74 (3H, s). ^{13}C NMR ($CDCl_3$, 100 MHz) δ_C 176.6, 155.1, 151.5, 109.2, 79.2, 58.4, 56.6, 55.6, 54.0, 50.9, 49.9, 49.3, 42.3, 40.9, 38.9, 38.9, 37.9, 36.9, 36.9, 36.9, 34.3, 33.0, 33.0, 33.0, 32.2, 32.2, 31.7, 30.6, 30.5, 28.1, 27.6, 26.6, 25.9, 25.8, 25.7, 25.7, 24.9, 24.9, 21.2, 21.2, 18.4, 16.4, 16.3, 15.5, 15.2. HRMS-ESI *m/z* [M+Na$^+$] calcd for $C_{43}H_{70}N_2O_3Na$: 685.5284; found 685.5288.

X-ray crystal data for **2g**: Single crystal X-ray diffraction data were collected at 296(2) K on a Bruker X8 APEXII KAPPA CCD diffractometer using MoKα radiation (λ = 0.71073 Å).

The structure was solved by intrinsic phasing method with SHELXTL XT (Bruker AXS 2014a) and refined using full-matrix least squares on F^2 with SHELXTL XLMP (Bruker AXS 2014b). $C_{43}H_{70}N_2O_3$, MW = 663.01, colorless, rod-like crystal: 0.04×0.12×0.26 mm^3, monoclinic space group $P2_1$, a = 13.319(7) Å, b = 10.417(5) Å, c = 14.441(7) Å, β = 108.481(10)°, V = 1900.4(17) $Å^3$, Z = 2, D_x = 1.159 g cm^{-3}, μ(Mo-Kα) = 0.071 mm^{-1}, F(000) = 732. Unique reflections: 7,008 (R_{int} = 0.2116). The final $R_1(F^2)$ = 0.0737 and $wR(F^2)$ = 0.1356 for 2,194 reflections with $F^2 > 2\ \sigma(F^2)$. Several crystallization attempts with different solvents did not give better quality single crystals. The small crystal chosen did not diffract X-ray well, giving rise to rather weak data. Crystal data of **2g** have been deposited with the Cambridge Crystallographic Data Centre (CCDC 1405981) and can be obtained free of charge via http://www.ccdc.cam.ac.uk/data_request/cif.

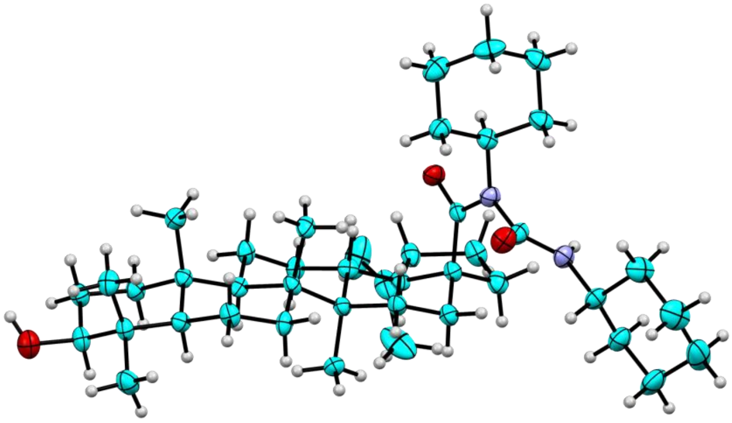

Figure 1S. ORTEP diagram of **2g**.

28-N-Cyclohexyl-N-[(cyclohexylamino)carbonyl]-3-O-acetyl betulinic acid (2h): Following the general procedure, reaction of **2g** (10.0 mg, 0.02 mmol), acetic anhydride (4 μL, 0.05 mmol), TEA (6 μL, 0.05 mmol), DMAP (trace amount) in CH_2Cl_2 (0.15 mL) after 1 h yielded **2h** (5.08 mg, 47.8%). Compound **2h**: White Gum; ^{1}H NMR ($CDCl_3$, 400 MHz) δ_H 5.42 (1H, d, J = 8.4 Hz), 4.72 (1H, s), 4.57 (1H, s), 4.46 (1H, m), 4.04 (1H, br), 3.70 (1H, br), 3.09 (1H, m), 2.33 (1H, m), 2.11 (1H, m), 2.03 (3H, s), 1.97 (1H, m), 1.96 (1H, m), 1.87 (1H, m), 1.86 (2H, m), 1.77 (1H, m), 1.68 (1H, m), 1.67 (3H, s), 1.66 (1H, m), 1.65 (1H, m), 1.63 (1H, m), 1.62 (1H, m), 1.61 (2H, m), 1.60 (2H, m), 1.58 (1H, m), 1.53 (1H, m), 1.51 (1H, m), 1.50 (1H, m), 1.49 (1H, m), 1.48 (1H, m), 1.44 (1H, m), 1.41 (2H, m), 1.34 (1H, m), 1.31 (1H, m), 1.30 (2H, m), 1.27 (1H, m), 1.26 (1H, m), 1.25 (1H, m), 1.22 (1H, m), 1.21 (2H, m,), 1.20 (1H, m), 1.18 (2H, m), 1.17 (1H, m), 1.14 (1H, m), 1.12 (1H, m), 0.96 (1H, m), 0.95 (3H, s), 0.93 (3H, s), 0.91 (1H, m), 0.86 (1H, m), 0.83 (6H, s), 0.82 (3H, s). ^{13}C

NMR ($CDCl_3$, 100 MHz) δ_C 175.5, 171.2, 155.3, 151.1, 109.5, 81.1, 58.4, 55.6, 53.9, 50.7, 49.9, 47.9, 47.8, 42.6, 41.1, 38.6, 37.9, 37.8, 37.2, 37.1, 34.3, 32.9, 32.9, 32.2, 32.2, 31.1, 30.6, 29.6, 28.2, 26.6, 25.9, 25.6, 25.0, 25.0, 23.9, 23.9, 23.9, 21.5, 21.3, 19.6, 18.3, 18.2, 16.3, 16.2, 5.2. ESI *m/z* [$M+Na^+$] calcd for $C_{45}H_{72}N_2O_4Na$: 727.5; found 727.5.

28-N-Cyclohexyl-N-[(cyclohexylamino)carbonyl]-3-O-propanoyl betulinic acid (2i): Following the general procedure, reaction of **2g** (10.0 mg, 0.02 mmol), propionic anhydride (6 µL, 0.05 mmol), TEA (6 µL, 0.05 mmol), DMAP (trace amount) in CH_2Cl_2 (0.15 mL) after 1 h yielded **2i** (5.32 mg, 49.1%). Compound **2h**: White Gum; ^1H NMR ($CDCl_3$, 400 MHz) δ_H 4.72 (1H, s), 4.58 (1H, s), 4.46 (1H, m), 3.42 (1H, m), 3.10 (1H, m), 2.31 (2H, m), 2.31 (1H, m), 2.16 (1H, m), 2.00 (1H, m), 1.89 (1H, m), 1.88 (1H, m), 1.87 (1H, m), 1.77 (1H, m), 1.67 (3H, s), 1.66 (1H, m), 1.65 (1H, m), 1.63 (1H, m), 1.62 (1H, m), 1.61 (3H, m), 1.60 (1H, m), 1.58 (3H, m), 1.57 (1H, m), 1.54 (1H, m), 1.50 (1H, m), 1.49 (2H, m), 1.47 (1H, m), 1.44 (1H, m), 1.42 (1H, m), 1.41 (1H, m), 1.36 (1H, m), 1.35 (1H, m), 1.31 (1H, m), 1.29 (1H, m), 1.26 (1H, m), 1.25 (1H, m), 1.22 (1H, m), 1.20 (1H, m), 1.18 (2H, m), 1.17 (1H, m), 1.14 (2H, m), 1.13 (3H, t, J = 8.0 Hz), 1.12 (1H, m), 1.10 (1H, m), 1.00 (1H, m), 0.96 (3H, s), 0.95 (3H, s), 0.93 (3H, s), 0.90 (3H, s), 0.89 (2H, m), 0.82 (3H, s). ^{13}C NMR ($CDCl_3$, 100 MHz) δ_C 175.3, 174.4, 155.2, 151.1, 109.7, 80.8, 56.6, 55.7, 54.0, 50.6, 49.9, 47.8, 47.1, 42.6, 41.0, 38.6, 38.0, 37.8, 37.3, 37.3, 34.3, 33.0, 33.0, 32.2, 32.2, 31.1, 30.6, 28.2, 28.1, 26.6, 25.9, 25.8, 25.8, 25.1, 25.1, 24.9, 23.9, 23.9, 21.1, 19.6, 18.3, 16.7, 16.3, 16.2, 14.8. ESI *m/z* [$M+Na^+$] calcd for $C_{46}H_{74}N_2O_4Na$: 741.5; found 741.5.

28-N-Cyclohexyl-N-[(cyclohexylamino)carbonyl]-3-O-butanoyl betulinic acid (2j): Following the general procedure, reaction of **2g** (10.0 mg, 0.02 mmol), butanoic anhydride (7 µL, 0.05 mmol), TEA (6 µL, 0.05 mmol), DMAP (trace amount) in CH_2Cl_2 (0.15 mL) after 1 h yielded **2j** (4.14 mg, 37.4%). Compound **2j**: White Gum; ^1H NMR ($CDCl_3$, 400 MHz) δ_H 4.72 (1H, s), 4.59 (1H, s), 4.47 (1H, m), 3.38 (1H, m), 3.07 (1H, m), 2.28 (2H, t, J = 8.0 Hz), 2.17 (1H, m), 2.02 (1H, m), 1.98 (2H, m), 1.93 (1H, m), 1.92 (1H, m), 1.78 (2H, m), 1.69 (3H, s), 1.64 (2H, m), 1.63 (1H, m), 1.62 (3H, m), 1.60 (1H, m), 1.59 (2H, m), 1.58 (2H, m), 1.57 (1H, m), 1.56 (1H, m), 1.53 (1H, m), 1.49 (1H, m), 1.48 (2H, m), 1.47 (1H, m), 1.46 (1H, m), 1.42 (2H, m), 1.35 (2H, m), 1.34 (1H, m), 1.26 (1H, m), 1.25 (1H, m), 1.21 (1H, m), 1.19 (1H, m), 1.18 (2H, m), 1.17 (2H, m), 1.15 (1H, m), 1.12 (2H, m), 1.10 (1H, m), 1.00 (1H, m), 0.97 (3H, s), 0.94 (3H, m), 0.93 (2H, m), 0.91 (3H, s), 0.85 (3H, s), 0.84 (3H, s), 0.83 (3H, s). ^{13}C NMR ($CDCl_3$, 100 MHz) δ_C 176.4, 173.8, 155.4, 151.4, 109.3, 80.8, 56.7, 55.7, 54.1, 50.8, 50.0, 49.9, 46.6, 42.3, 40.9, 38.6, 38.0, 37.8, 37.3, 36.9, 35.4, 34.4, 33.4,

32.4, 32.3, 32.3, 31.5, 30.6, 29.7, 28.1, 26.6, 26.6, 25.9, 25.9, 25.7, 25.0, 25.0, 22.4, 21.2, 19.6, 18.4, 18.3, 18.3, 16.4, 16.2, 15.2, 14.1. ESI *m/z* [M+Na$^+$] calcd for $C_{47}H_{77}N_2O_4Na$: 755.5; found 755.5.

28-N-Cyclohexyl-N-[(cyclohexylamino)carbonyl]-3-O-pentanoyl betulinic acid (2k): Following the general procedure, reaction of **2g** (10.0 mg, 0.02 mmol), valeric anhydride (9 µL, 0.05 mmol), TEA (6 µL, 0.05 mmol), DMAP (trace amount) in CH_2Cl_2 (0.15 mL) after 1 h yielded **2k** (4.36 mg, 38.7%). Compound **2k**: White Gum; ^{1}H NMR ($CDCl_3$, 400 MHz) δ_H 4.72 (1H, s), 4.58 (1H, s), 4.46 (1H, m), 3.08 (1H, m), 3.05 (1H, m), 2.47 (2H, m), 2.29 (1H, m), 2.19 (1H, m), 2.19 (1H, m), 1.93 (1H, m), 1.90 (1H, m), 1.82 (1H, m), 1.75 (1H, m), 1.74 (1H, m), 1.69 (3H, s), 1.68 (1H, m), 1.64 (1H, m), 1.64 (1H, m), 1.62 (2H, m), 1.61 (2H, m), 1.60 (1H, m), 1.60 (2H, m), 1.59 (1H, m), 1.58 (1H, m), 1.56 (2H, m), 1.55 (1H, m), 1.48 (3H, m), 1.45 (1H, m), 1.40 (2H, m), 1.35 (3H, m), 1.34 (1H, m), 1.33 (2H, m), 1.32 (1H, m,), 1.30 (2H, m), 1.28 (1H, m), 1.25 (3H, m), 1.21 (1H, m), 1.18 (1H, m), 1.18 (2H, m), 1.15 (2H, m,), 1.12 (1H, m), 1.09 (1H, m), 0.97 (3H, s), 0.96 (3H, s), 0.96 (1H, m), 0.91 (3H, t, *J* = 8.0 Hz, H-5′), 0.89 (3H, s), 0.84 (3H, s), 0.83 (3H, s). ^{13}C NMR ($CDCl_3$, 100 MHz) δ_C 155.4, 151.4, 176.8, 173.8, 109.3, 80.7, 58.3, 55.6, 53.9, 50.8, 50.0, 49.8, 46.6, 42.3, 41.0, 38.5, 38.0, 37.8, 37.3, 36.9, 34.7, 34.4, 33.0, 33.0, 32.2, 32.2, 31.7, 30.5, 30.5, 28.1, 26.7, 26.6, 25.9, 25.7, 24.9, 24.9, 23.9, 23.9, 23.9, 22.4, 21.8, 19.7, 18.3, 16.7, 16.3, 16.2, 14.8, 13.8. ESI *m/z* [M+Na]$^+$ calcd for $C_{48}H_{78}N_2O_4$: 769.6; found 769.6.

28-N-Cyclohexyl-N-[(cyclohexylamino)carbonyl]-3-O-hexanoyl betulinic acid (2l): Following the general procedure, reaction of **2g** (10.0 mg, 0.02 mmol), hexanoic anhydride (10 µL, 0.05 mmol), TEA (6 µL, 0.05 mmol), DMAP (trace amount) in CH_2Cl_2 (0.15 mL) after 1 h yielded **2** (2.06 mg, 17.9%). Compound **2l**: White Gum; ^{1}H NMR ($CDCl_3$, 400 MHz) δ_H 5.40 (1H, d, *J* = 6.0 Hz, NH), 4.73 (1H, s,), 4.60 (1H, s), 4.47 (1H, m), 4.04 (1H, br), 3.70 (1H, br), 3.10 (1H, m), 2.42 (1H, m), 2.30 (2H, t, *J* = 7.4 Hz, H-2′), 2.04 (1H, m), 1.97 (1H, m), 1.91 (1H, m), 1.90 (1H, m), 1.85 (2H, m), 1.77 (2H, m), 1.69 (3H, s), 1.68 (2H, m), 1.66 (1H, m), 1.65 (2H, m), 1.62 (2H, m), 1.61 (2H, m), 1.59 (1H, m), 1.58 (2H, m), 1.57 (2H, m), 1.56 (2H, m), 1.55 (2H, m), 1.54 (2H, m), 1.49 (3H, m), 1.44 (2H, m), 1.41 (1H, m), 1.37 (1H, m), 1.36 (2H, m), 1.35 (1H, m), 1.34 (2H, m), 1.33 (2H, m), 1.27 (1H, m), 1.26 (1H, m), 1.22 (2H, m), 1.21 (3H, m), 1.20 (2H, m), 1.19 (1H, m), 1.18 (2H, m), 1.17 (2H, m), 1.16 (2H, m), 1.14 (1H, m), 1.12 (1H, m), 0.97 (3H, s), 0.97 (1H, m), 0.95 (3H, s), 0.91 (2H, m), 0.90 (3H, m), 0.86 (2H, m), 0.85 (3H, s), 0.83 (6H, s). ^{13}C NMR ($CDCl_3$, 100 MHz) δ_C 182.6, 173.7, 153.2, 151.0, 110.0, 80.6, 58.3, 55.3, 53.4, 50.6, 49.0, 48.8, 47.8, 42.7, 41.1,

38.6, 38.0, 37.8, 37.3, 37.3, 34.8, 34.4, 33.0, 33.0, 32.2, 32.0, 31.2, 30.6, 30.0, 28.1, 27.8, 26.7, 26.7, 25.7, 25.0, 24.9, 24.9, 24.0, 24.0, 23.9, 22.6, 21.3, 19.9, 18.4, 17.1, 16.3, 16.2, 14.8, 14.0. ESI *m/z* $[M+H]^+$ calcd for $C_{49}H_{81}N_2O_4$: 761.6; found 761.6.

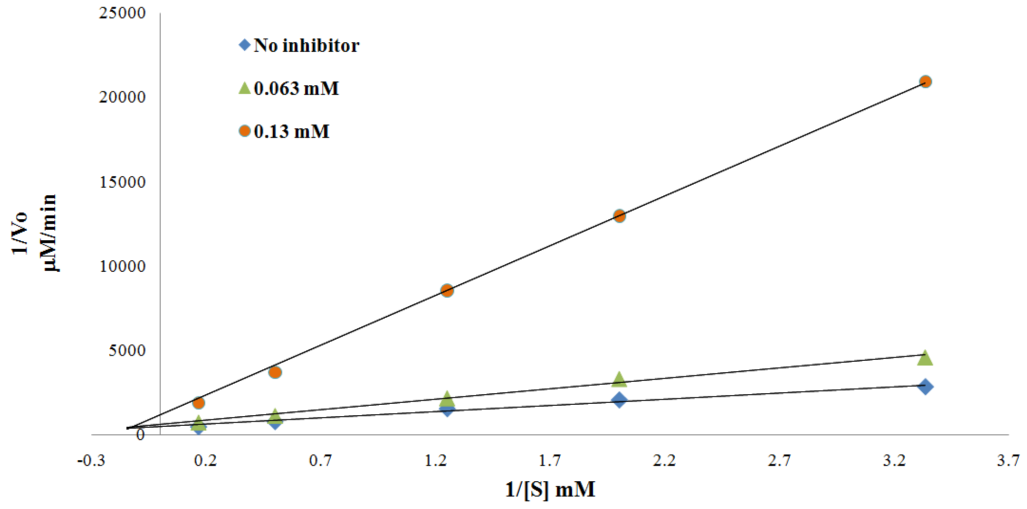

Figure 2S. Lineweaver-Burk plots for inhibitory activity of compound **2** against yeast *α*-glucosidase.

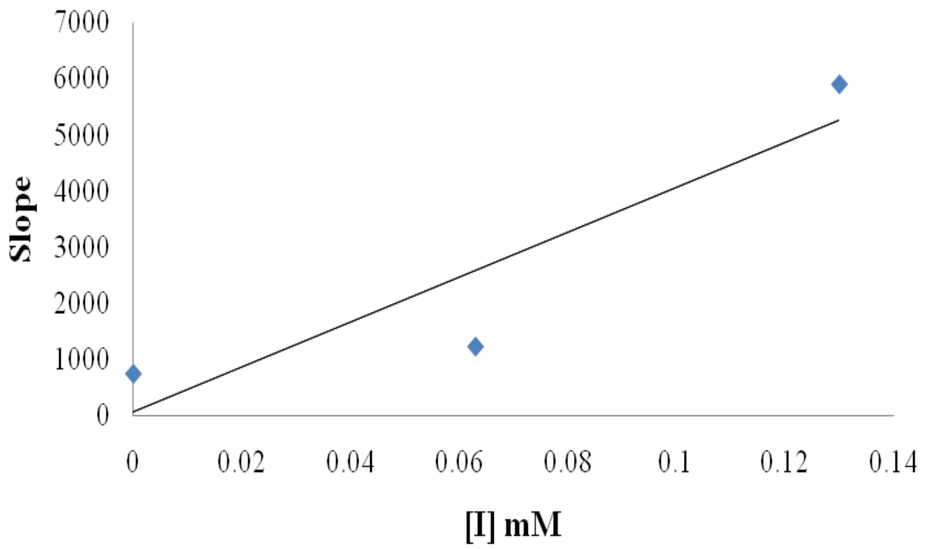

Figure 3S. Secondary plot of slope *vs* [I] for deduction of K_i of compound **2** against yeast *α*-glucosidase.

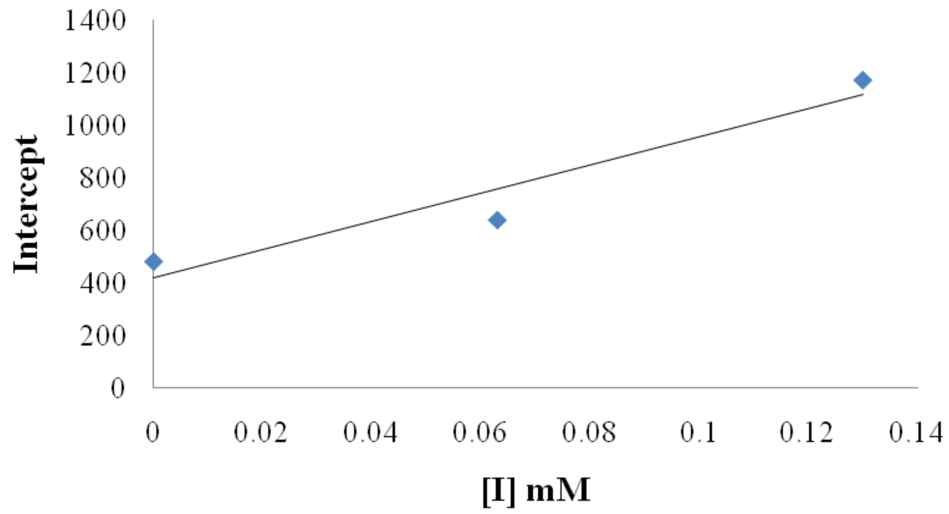

Figure 4S. Secondary plot of intercept *vs* [I] for deduction of K'_i of compound **2** against yeast α-glucosidase.

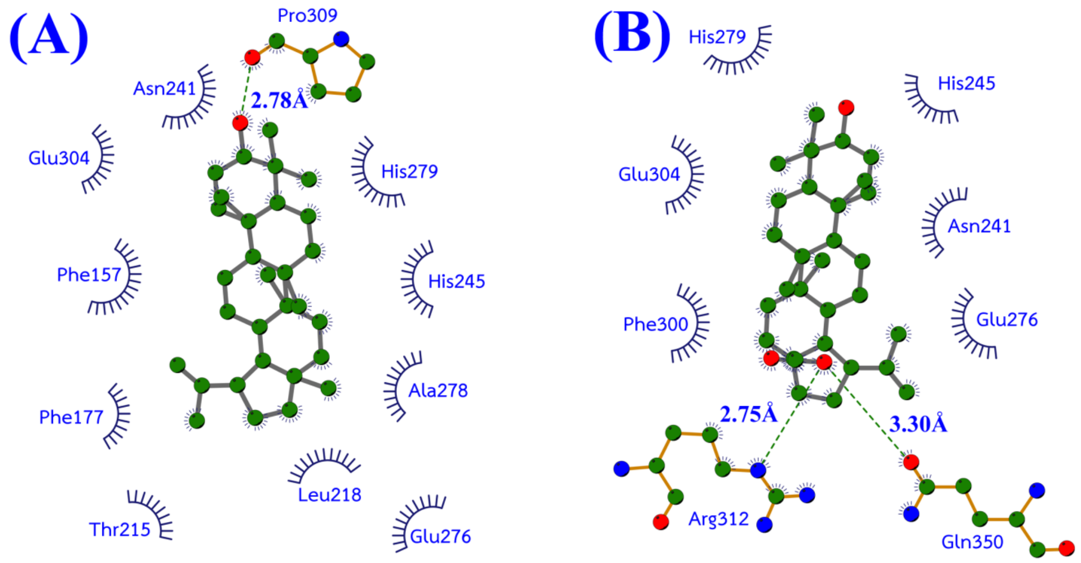

Figure 5S. The hydrophobic analysis of compounds **1** (A) and **2** (B). The H-bond interactions were shown as green dotted lines and the hydrophobic interactions with surrounding amino acids are shown as arcs with spokes radiating towards the inhibitor. These schematic diagrams were generated using LigPlot+ software (Laskowski & Swindells, 2011).

References

Ahmad FBH, Moghaddam MG, Basri M, Abdul Rahman MB. 2010. Anticancer activity of 3-O-acylated betulinic acid derivatives obtained by enzymatic synthesis. Biosci Biotechnol Biochem. 74:1025-1029.

De Le Silva M, David JP, Silva LCRC, Santos RAF, David JM, Lima LS, Reis PS, Fontana R. 2012. Bioactive oleanane, lupane and ursane triterpene acid derivatives. Molecules. 17: 12197-12205.

Zhang SJ, Ge QF, Guo DW, Hu WX, Liu HZ. 2010. Synthesis and anticancer evaluation of α-lipoic acid derivatives. Bioorg Med Chem Lett. 20:3078-3083.

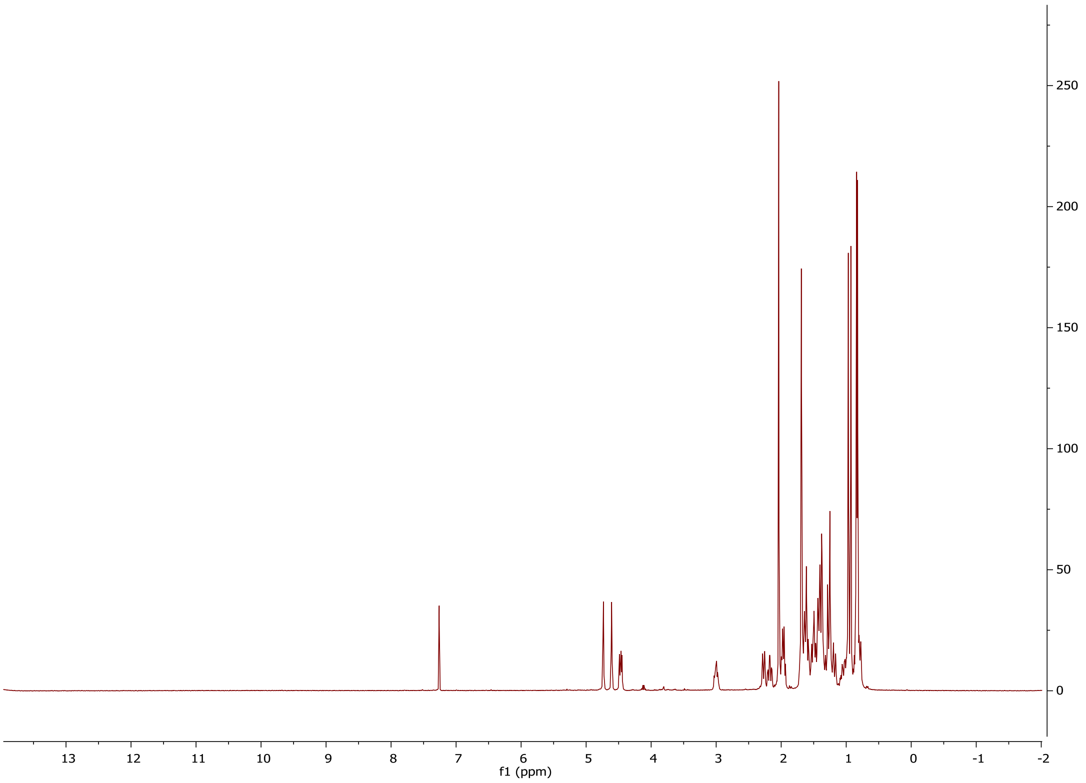

Figure 6S. ^{1}H NMR spectrum of 3-*O*-acetyl betulinic acid (**2a**) in $CDCl_3$

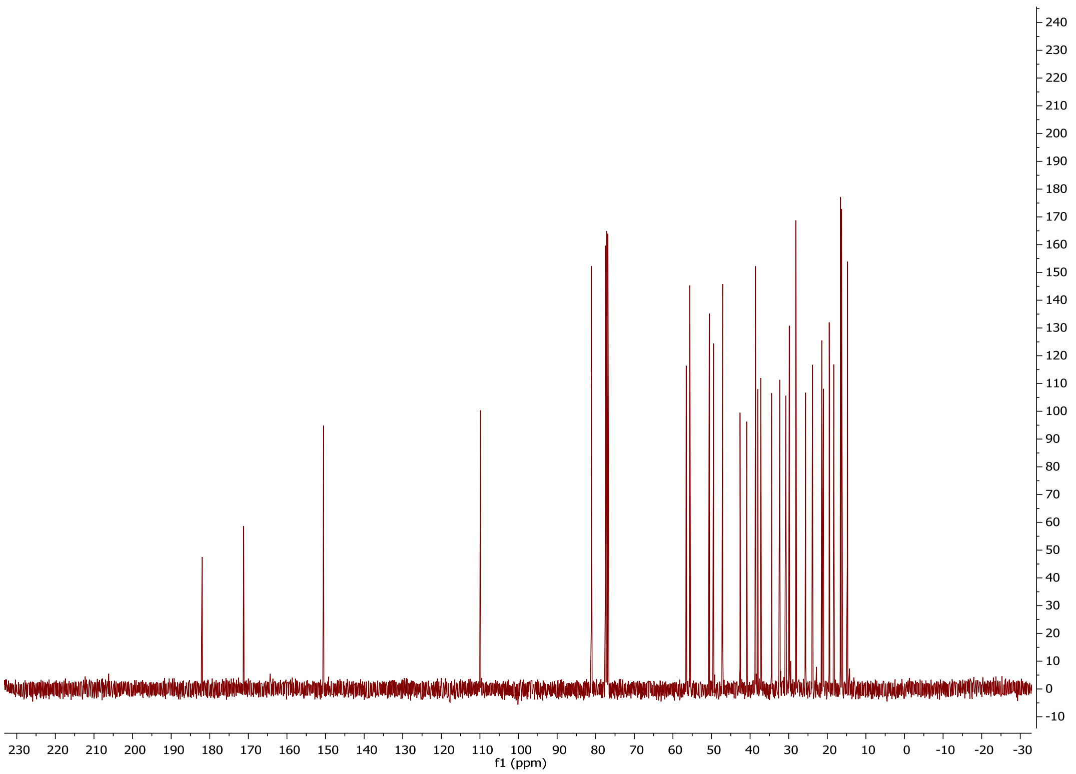

Figure 7S. ^{13}C NMR spectrum of 3-*O*-acetyl betulinic acid (**2a**) in $CDCl_3$

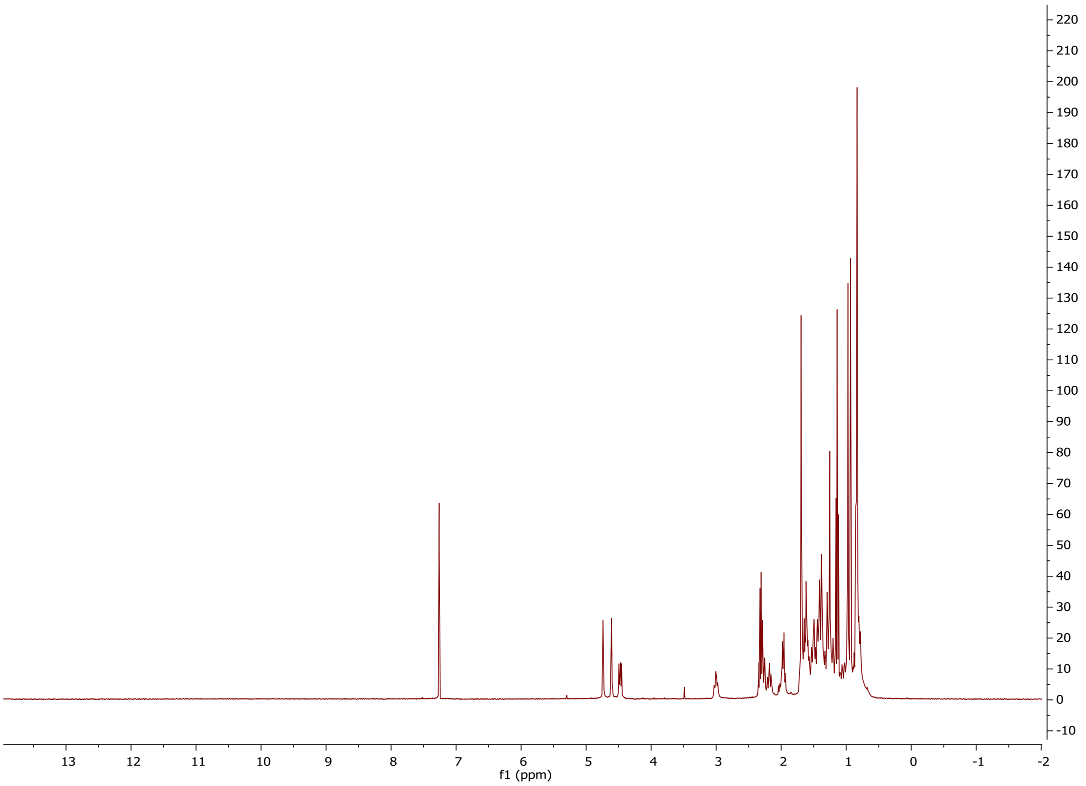

Figure 8S. ^{1}H NMR spectrum of 3-*O*-propanoybetulinic acid (**2b**) in $CDCl_3$

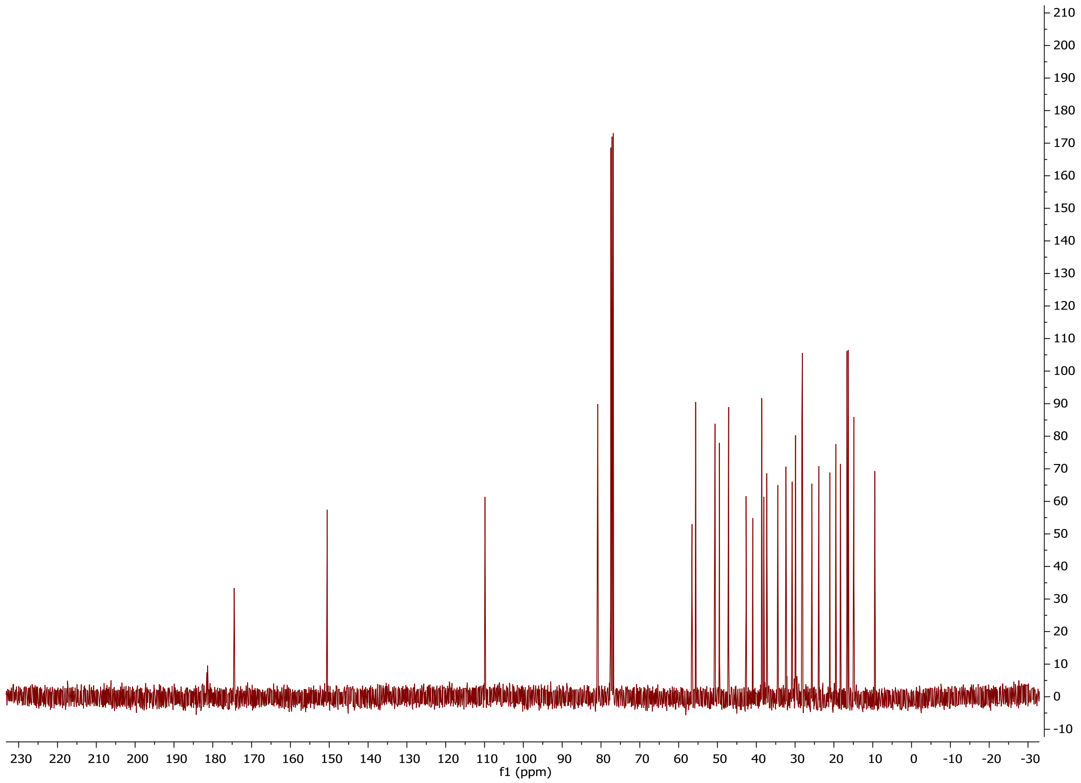

Figure 9S. ^{13}C NMR spectrum of 3-*O*-propanoyl betulinic acid (**2b**) in $CDCl_3$

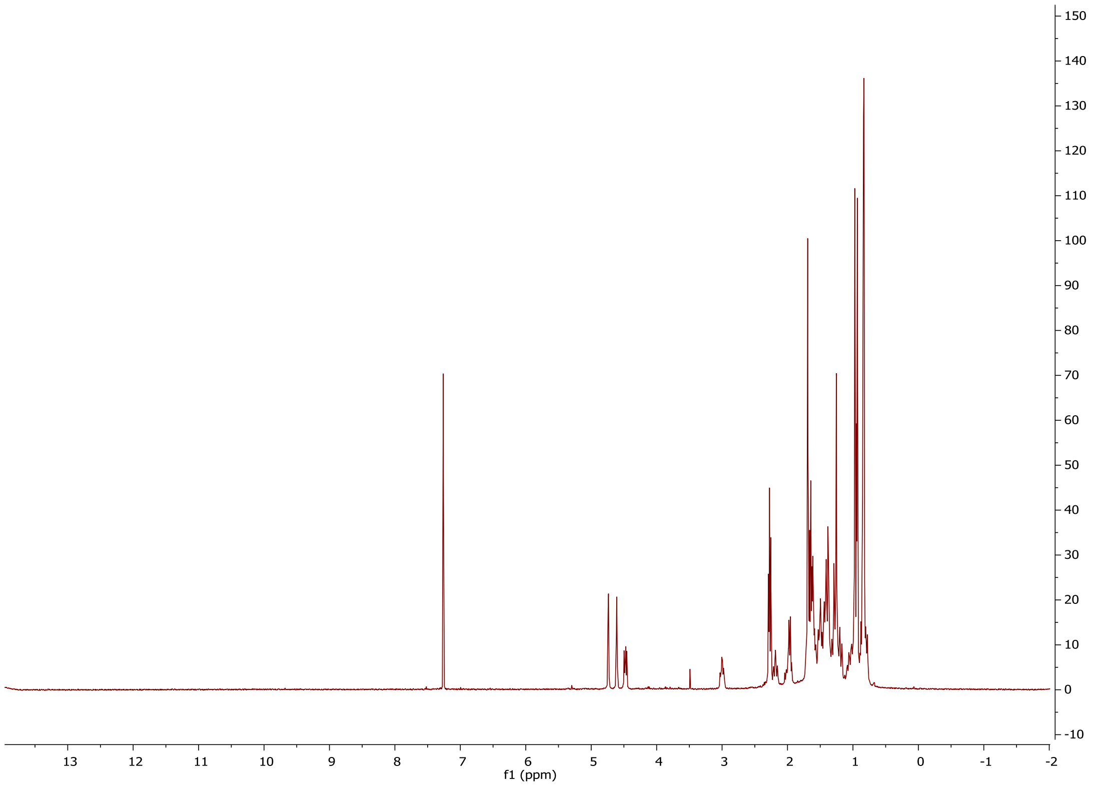

Figure 10S. ^{1}H NMR spectrum of 3-*O*-butanoyl betulinic acid (**2c**) in $CDCl_3$

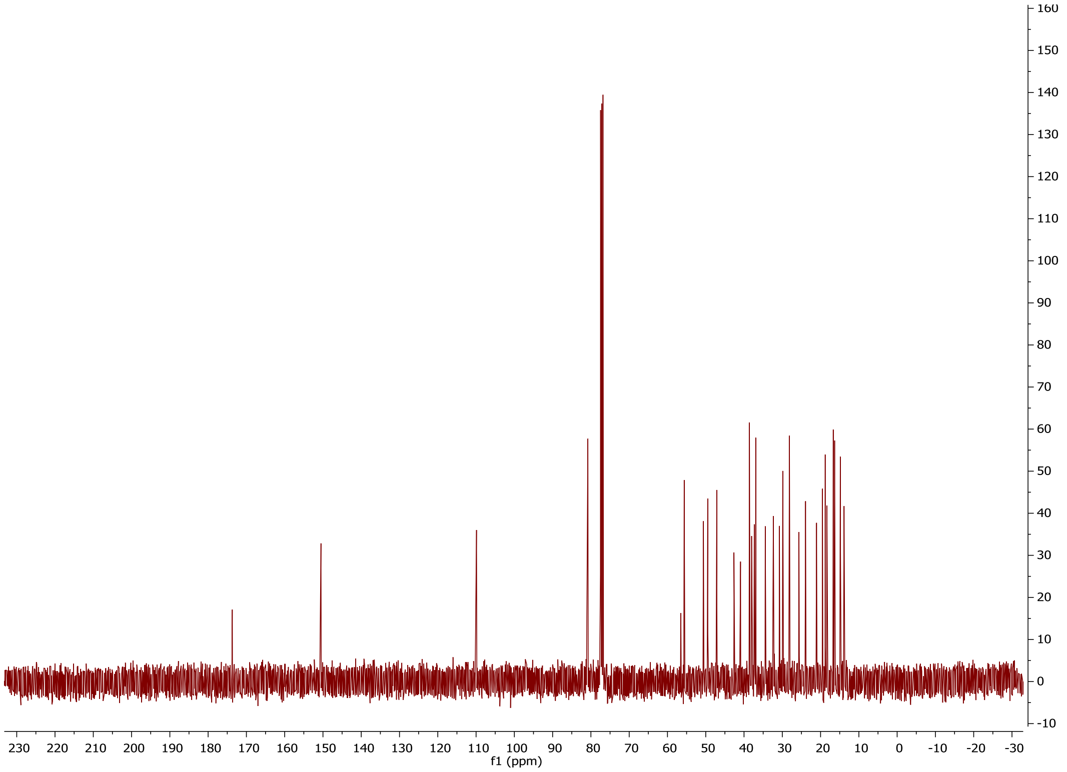

Figure 11S. ^{13}C NMR spectrum of 3-*O*-butanoyl betulinic acid (**2c**) in $CDCl_3$

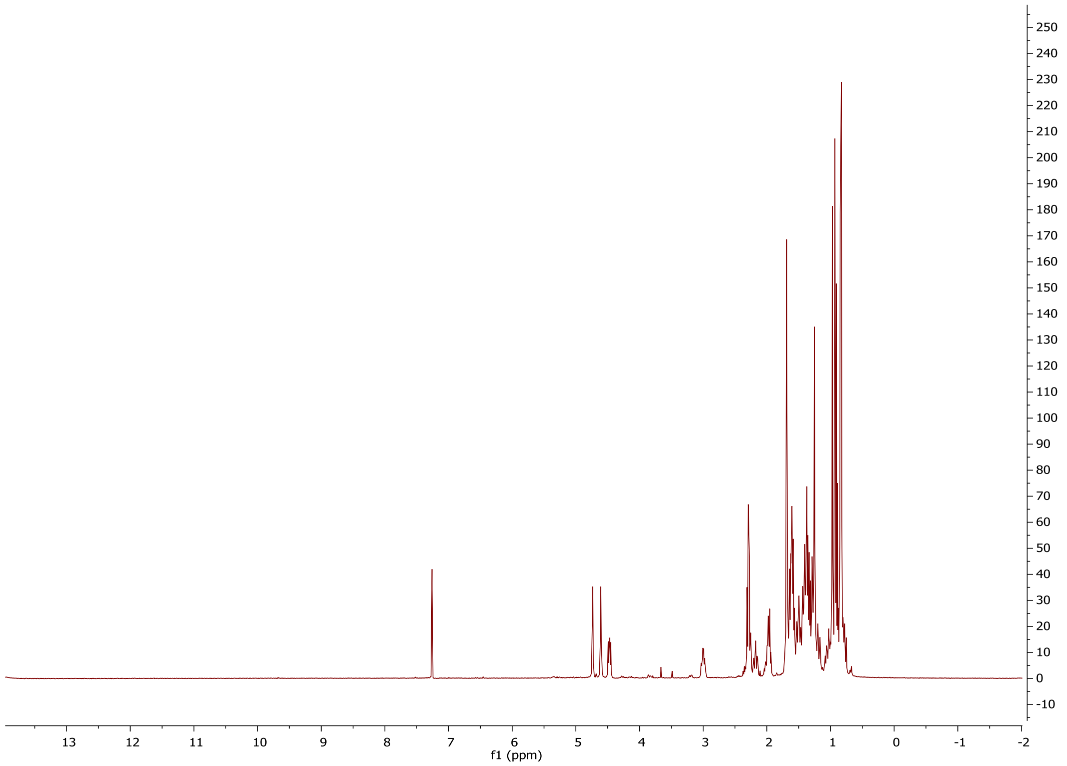

Figure 12S. ^{1}H NMR spectrum of 3-*O*-pentanoyl betulinic acid (**2d**) in $CDCl_3$

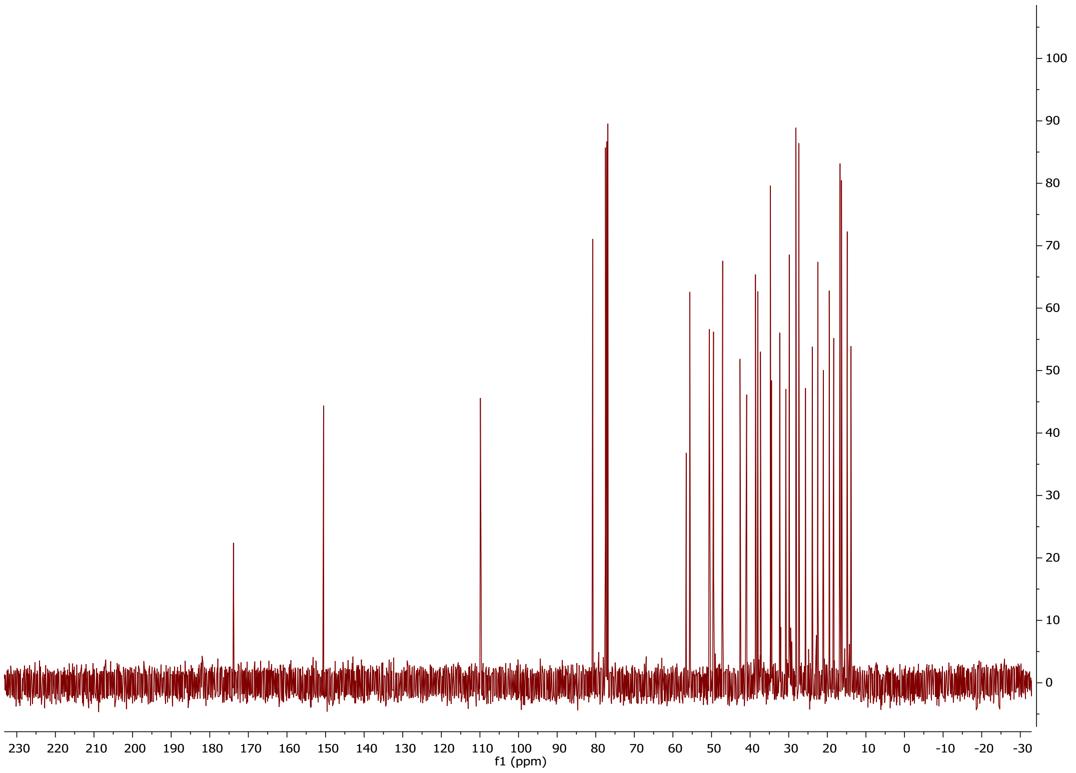

Figure 13S. ^{13}C NMR spectrum of 3-*O*-pentanoyl betulinic acid (**2d**) in $CDCl_3$

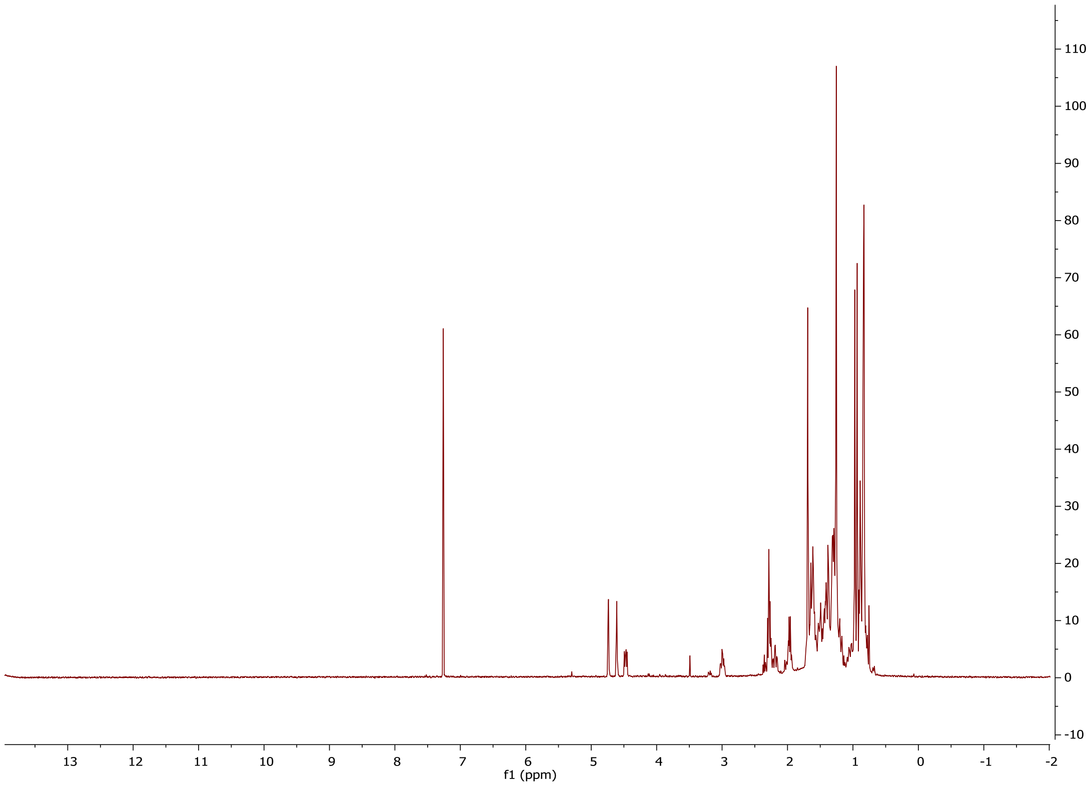

Figure 14S. ^{1}H NMR spectrum of 3-*O*-hexanoyl betulinic acid in(**2e**) $CDCl_3$

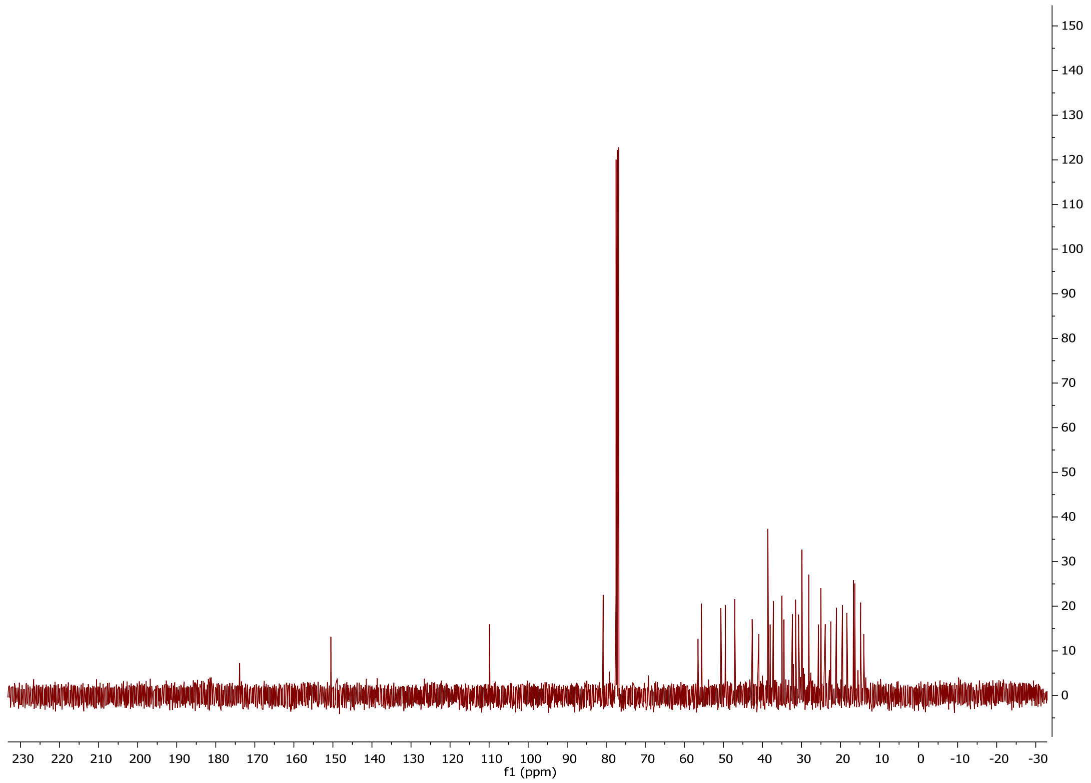

Figure 15S. ^{13}C NMR spectrum of 3-*O*-hexanoyl betulinic acid (**2e**) in $CDCl_3$

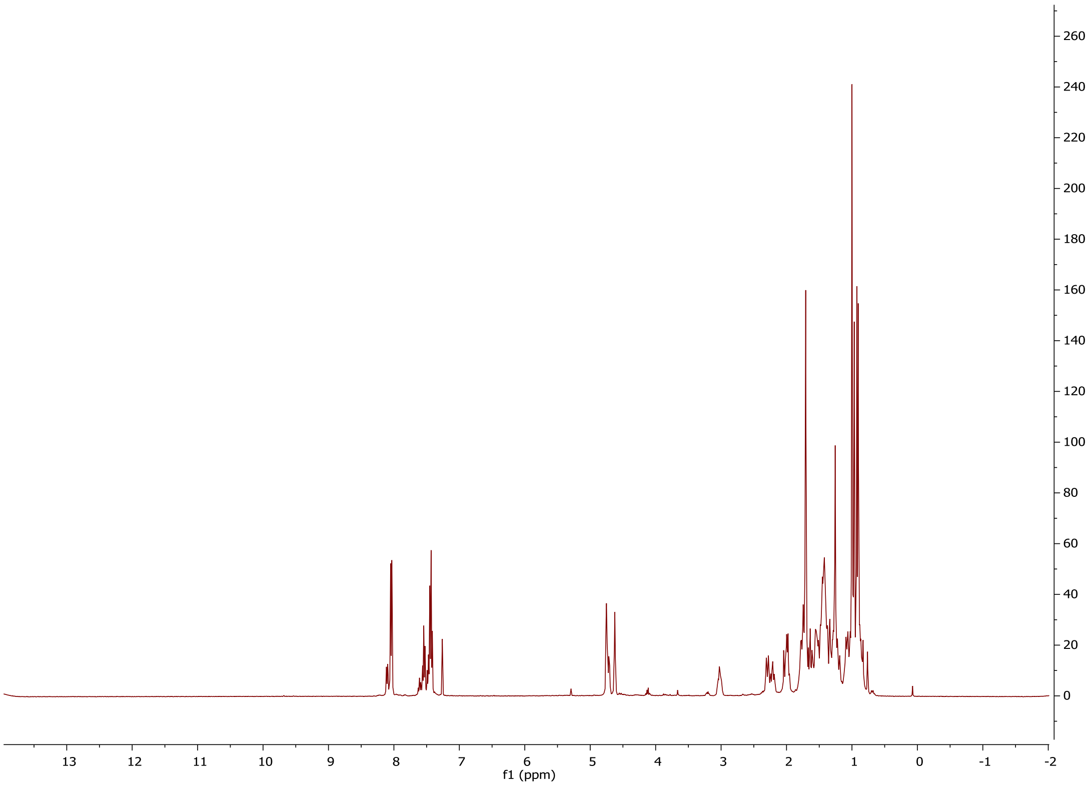

Figure 16S. ^{1}H NMR spectrum of 3-*O*-benzoyl betulinic acid (**2f**) in $CDCl_3$

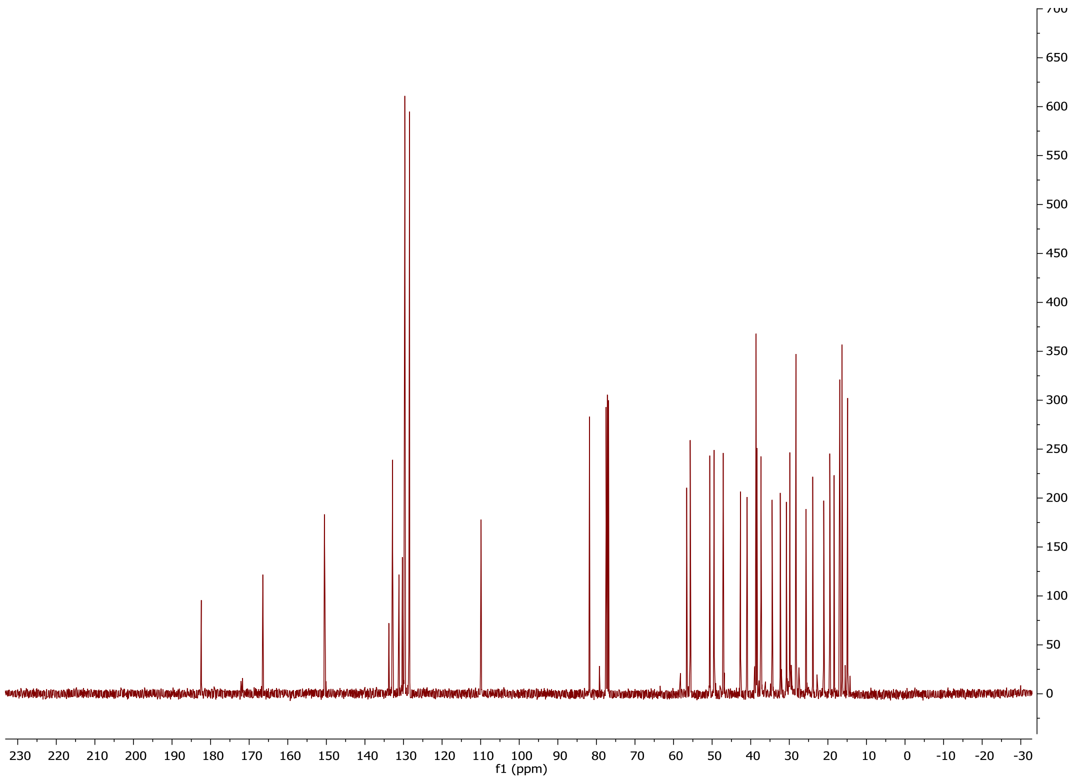

Figure 17S. ^{13}C NMR spectrum of 3-*O*-benzoyl betulinic acid (**2f**) in $CDCl_3$

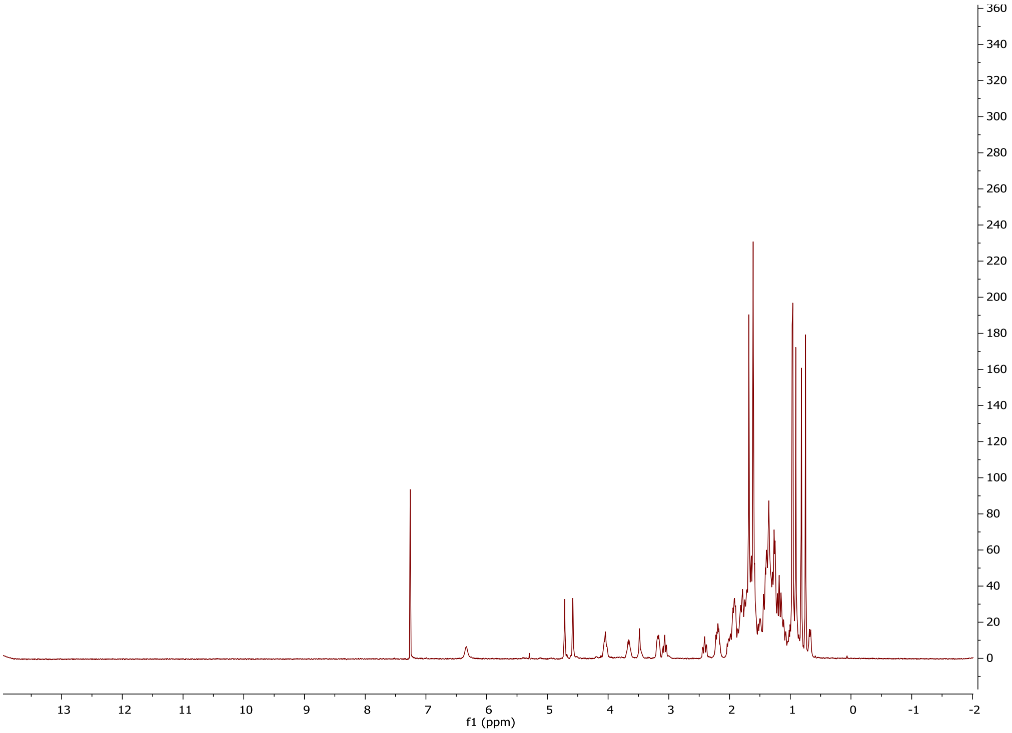

Figure 18S. ^{1}H NMR spectrum of 28-(cyclohexylamino)-cyclohexylimino-methanebetulinic acid (**2g**) in $CDCl_3$

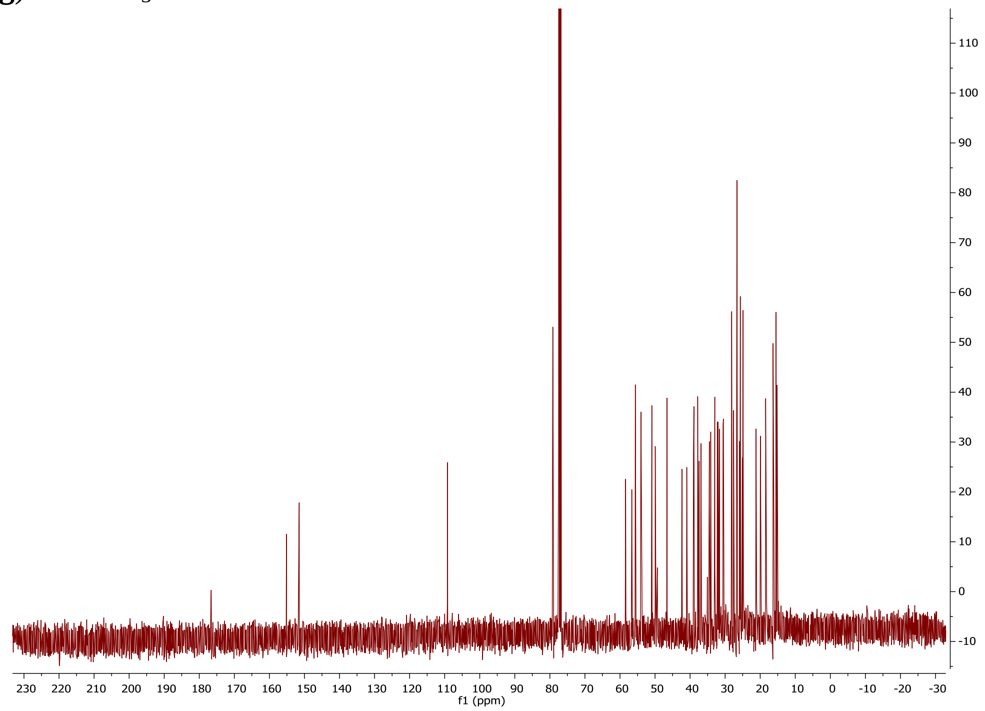

Figure 19S. ^{13}C NMR spectrum of 28-(cyclohexylamino)-cyclohexylimino-methanebetulinic acid (**2g**) in $CDCl_3$

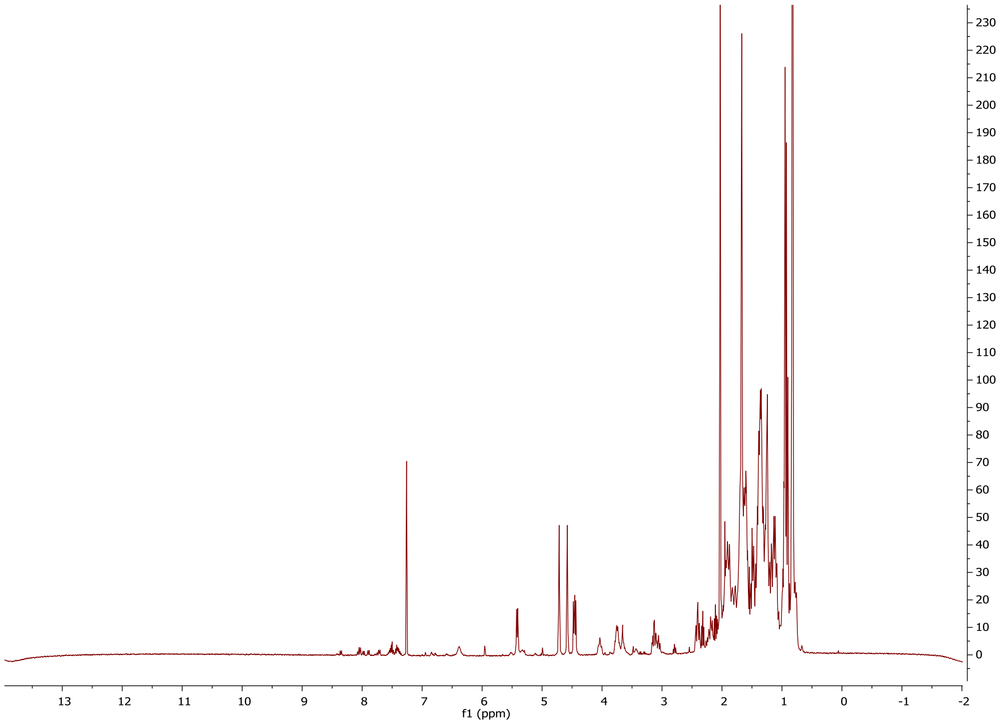

Figure 20S. ^{1}H NMR spectrum of 28-(cyclohexylamino)-cyclohexylimino-methane-3-*O*-acetyl betulinic acid (**2h**) in $CDCl_3$

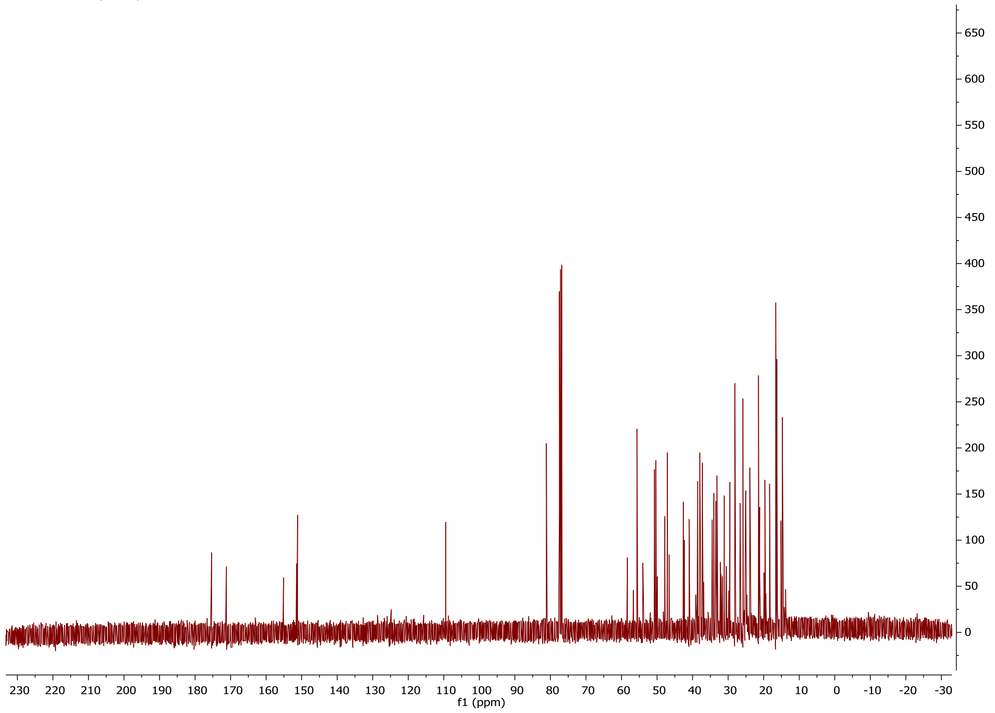

Figure 21S. ^{13}C NMR spectrum of 28-(cyclohexylamino)-cyclohexylimino-methane-3-*O*-acetyl betulinic acid (**2h**) in $CDCl_3$

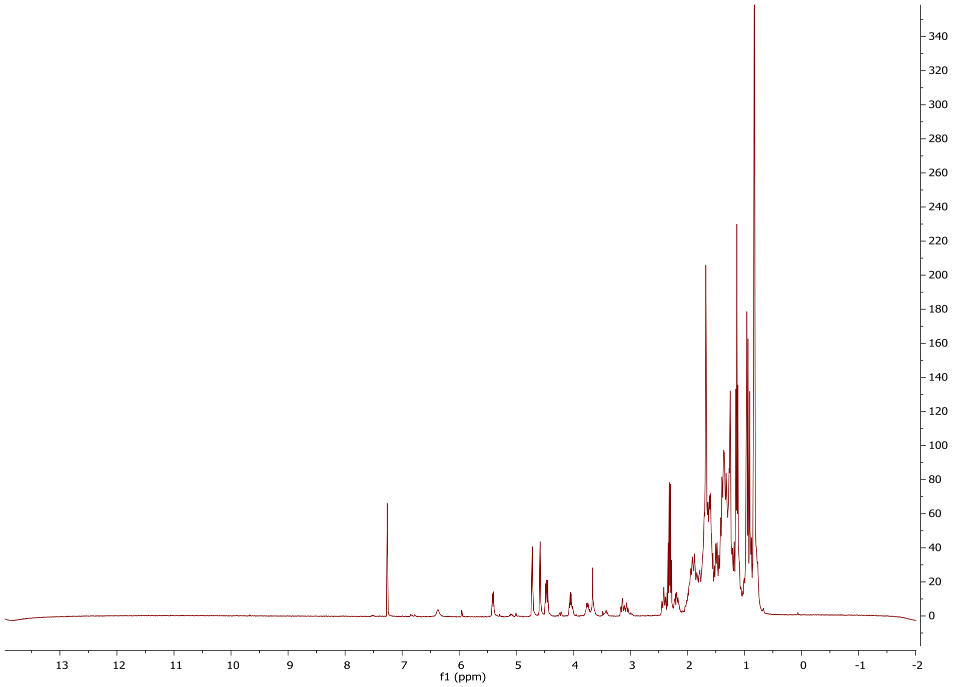

Figure 22S. ^{1}H NMR spectrum of 28-(cyclohexylamino)-cyclohexylimino-methane-3-*O*-propanoyl betulinic acid (**2i**) in $CDCl_3$

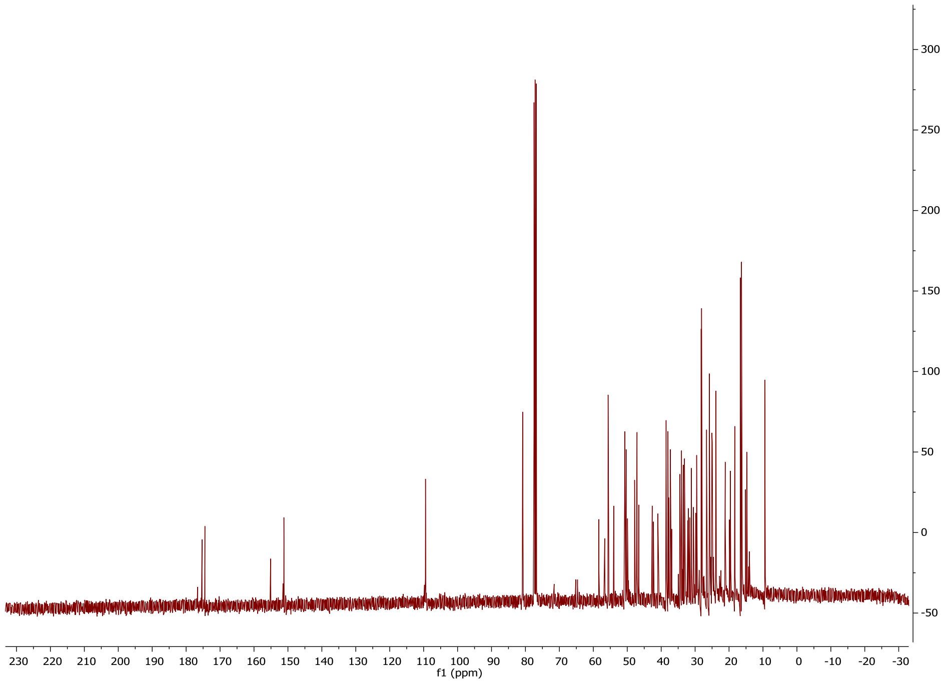

Figure 23S. ^{13}C NMR spectrum of 28-(cyclohexylamino)-cyclohexylimino-methane-3-*O*-propanoyl betulinic acid (**2i**) in $CDCl_3$

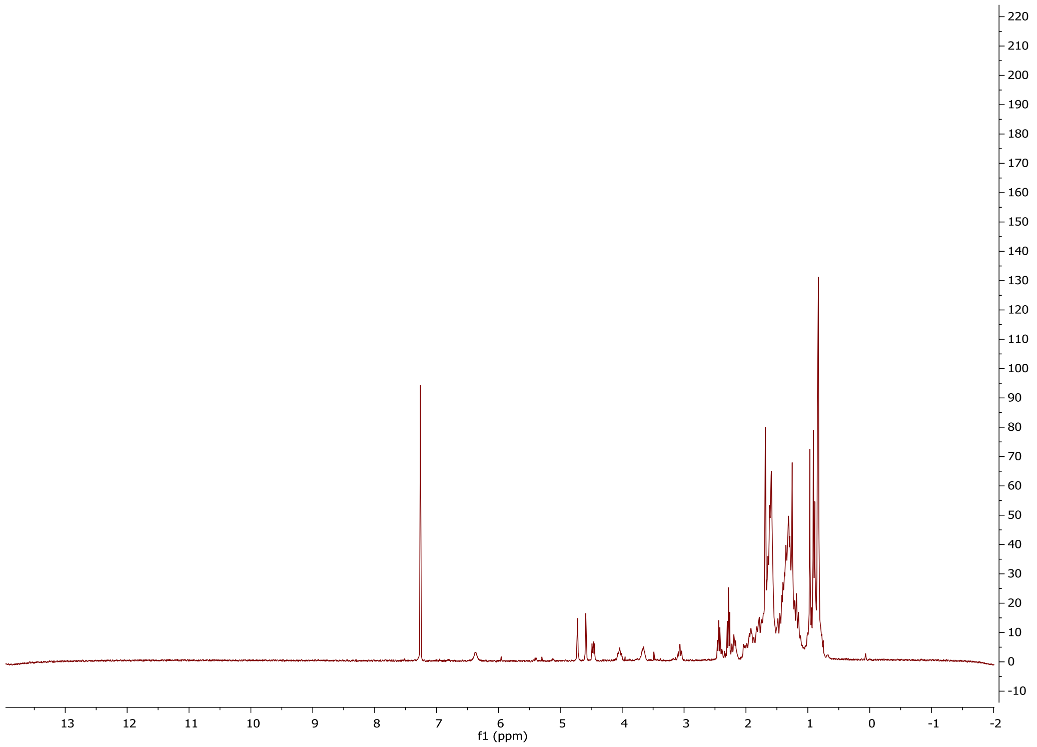

Figure 24S. ^{1}H NMR spectrum of 28-(cyclohexylamino)-cyclohexylimino-methane-3-*O*-butanoyl betulinic acid (**2j**) in $CDCl_3$

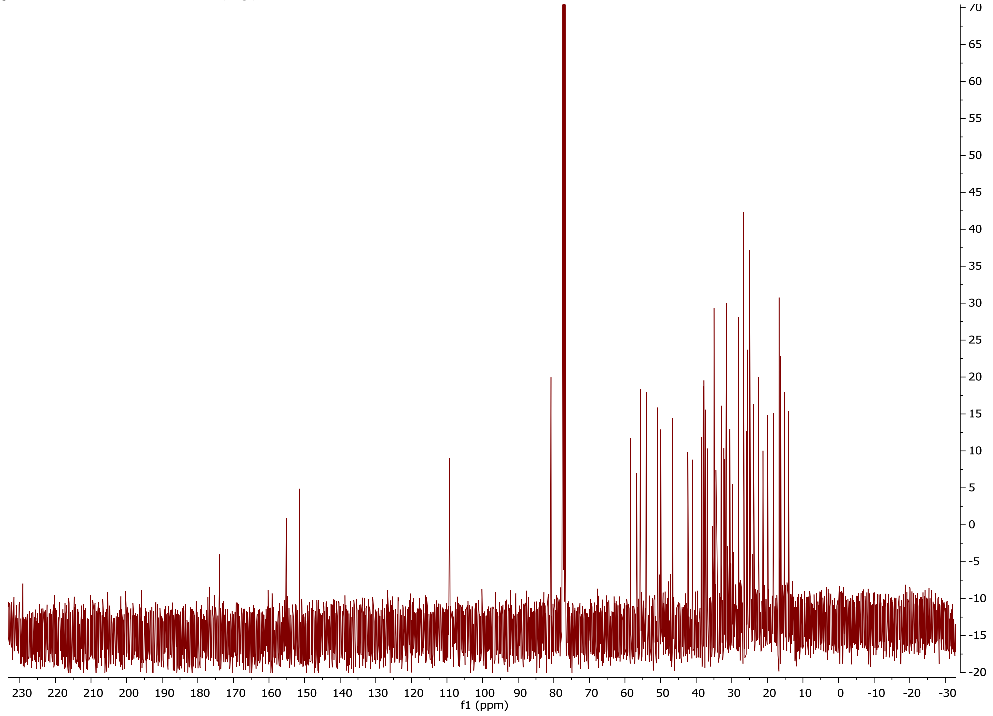

Figure 25S. ^{13}C NMR spectrum of 28-(cyclohexylamino)-cyclohexylimino-methane-3-*O*-butanoyl betulinic acid (**2j**) in $CDCl_3$

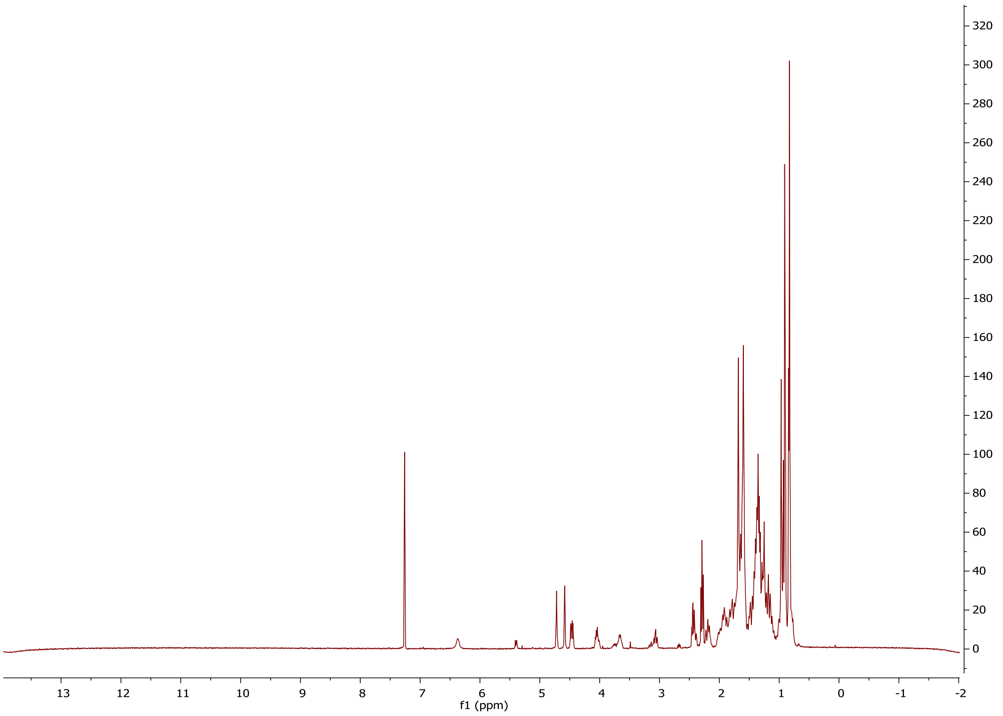

Figure 26S. ^{1}H NMR spectrum of 28-(cyclohexylamino)-cyclohexylimino-methane-3-*O*-pentanoyl betulinic acid (**2k**) in $CDCl_3$

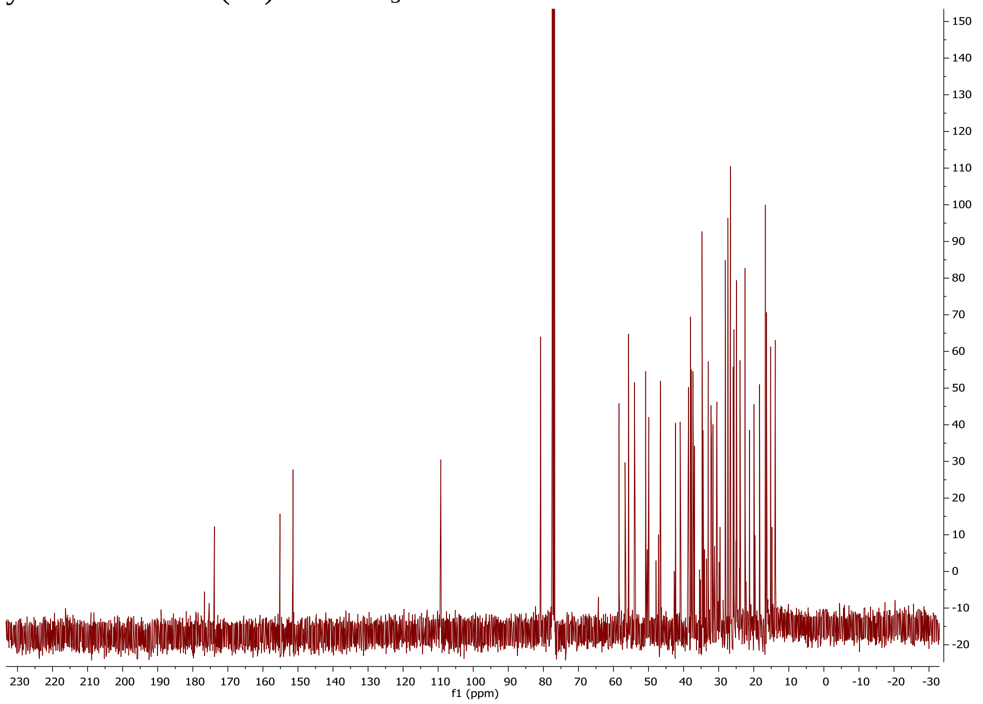

Figure 27S. ^{13}C NMR spectrum of 28-(cyclohexylamino)-cyclohexylimino-methane-3-*O*-pentanoyl betulinic acid (**2k**) in $CDCl_3$

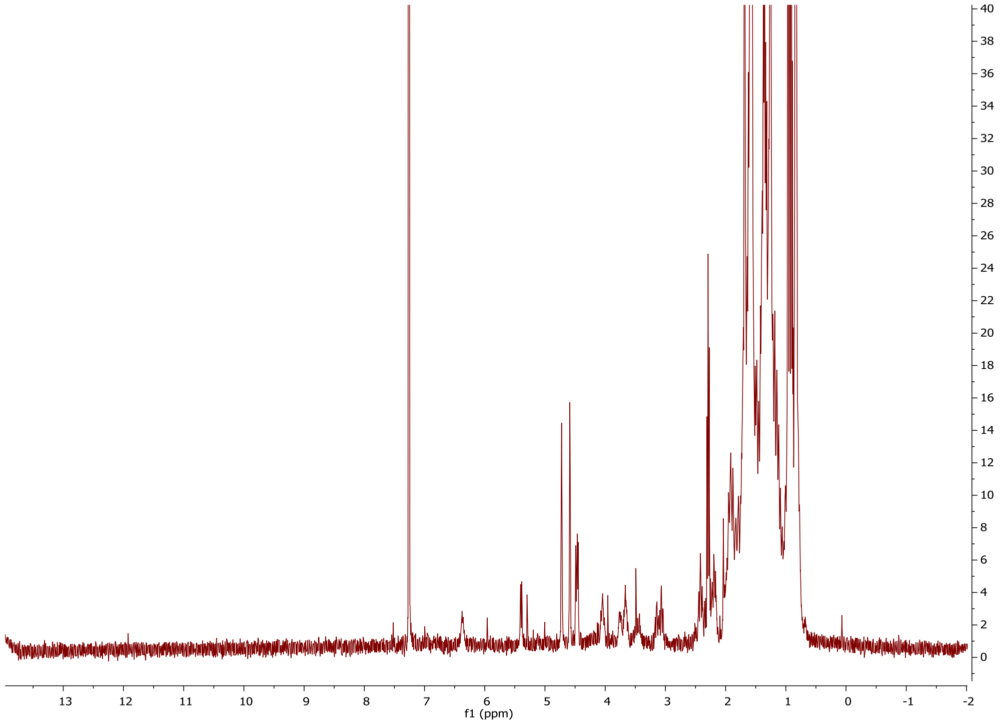

Figure 28S. ^{1}H NMR spectrum of 28-(cyclohexylamino)-cyclohexylimino-methane-3-*O*-hexanoyl betulinic acid (**2l**) in $CDCl_3$

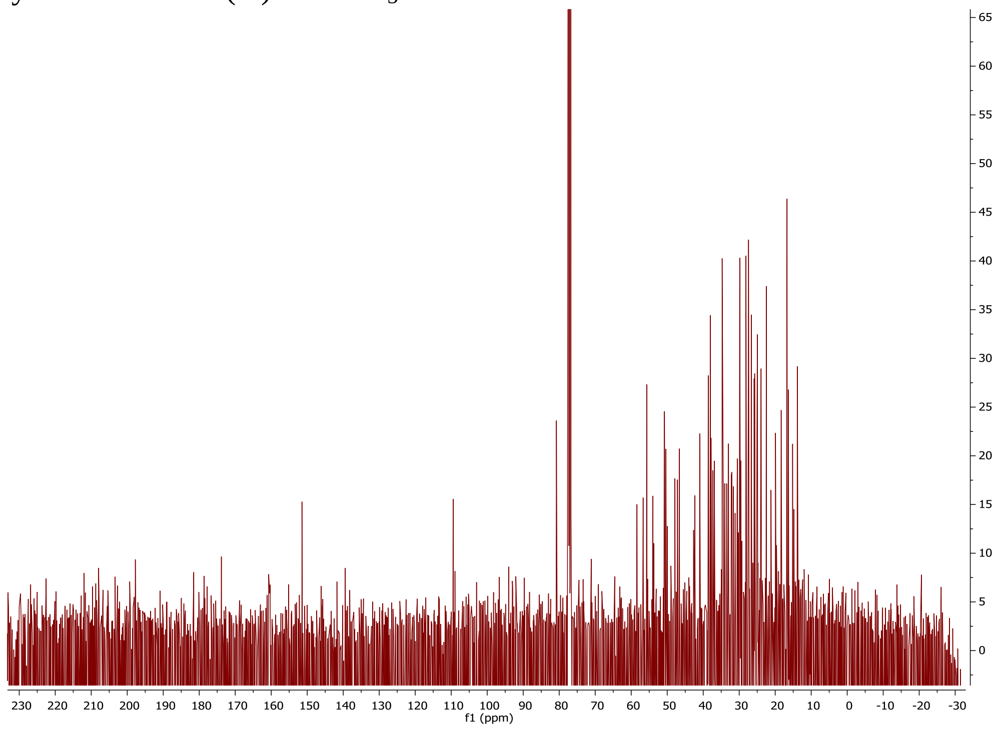

Figure 29S. ^{13}C NMR spectrum of 28-(cyclohexylamino)-cyclohexylimino-methane-3-*O*-hexanoyl betulinic acid (**2l**) in $CDCl_3$

Table 1S. Comparison the DOPE score obtained from homology modeling approach[a].

Model	DOPE score
Gi_411229_emb_CAA00532.1.BL00010002	-40039.8
Gi_411229_emb_CAA00532.1.BL00010010	-39861.7
Gi_411229_emb_CAA00532.1.BL00010007	-39815.3
Gi_411229_emb_CAA00532.1.BL00010009	-39744.6
Gi_411229_emb_CAA00532.1.BL00010006	-39664.4
Gi_411229_emb_CAA00532.1.BL00010005	-39477.1
Gi_411229_emb_CAA00532.1.BL00010008	-39216.4
Gi_411229_emb_CAA00532.1.BL00010003	-38875.7
Gi_411229_emb_CAA00532.1.BL00010001	-38839.4
Gi_411229_emb_CAA00532.1.BL00010004	-38680.2

[a] The structure which showed a lowest DOPE score was selected for further study.
